# Supplementary material for: Preimmunization correlates of protection shared across malaria vaccine trials in adults
Source: NPJ Vaccines. 2022 Jan 14;7:5. doi: 10.1038/s41541-021-00425-1 (PMC8760258; doi:10.1038/s41541-021-00425-1)
Supplement: Supplementary file 1 — Supplementary Information [file 41541_2021_425_MOESM1_ESM.pdf]

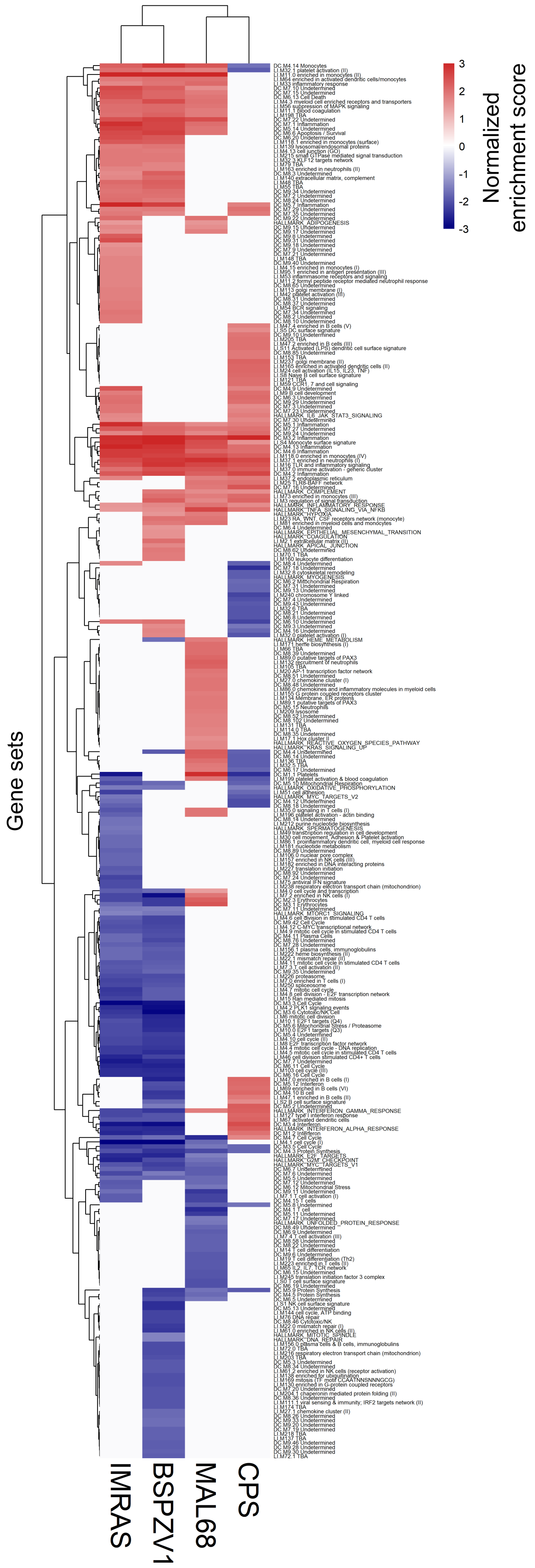

Supplementary Figure 1. Heatmap of GSEA normalized enrichment scores for protection-associated gene sets in each of the four malaria vaccine trials analyzed. Gene sets shown are those with a significant enrichment score in at least one trial. For visualization purposes, non-significant normalized enrichment scores were set to zero.

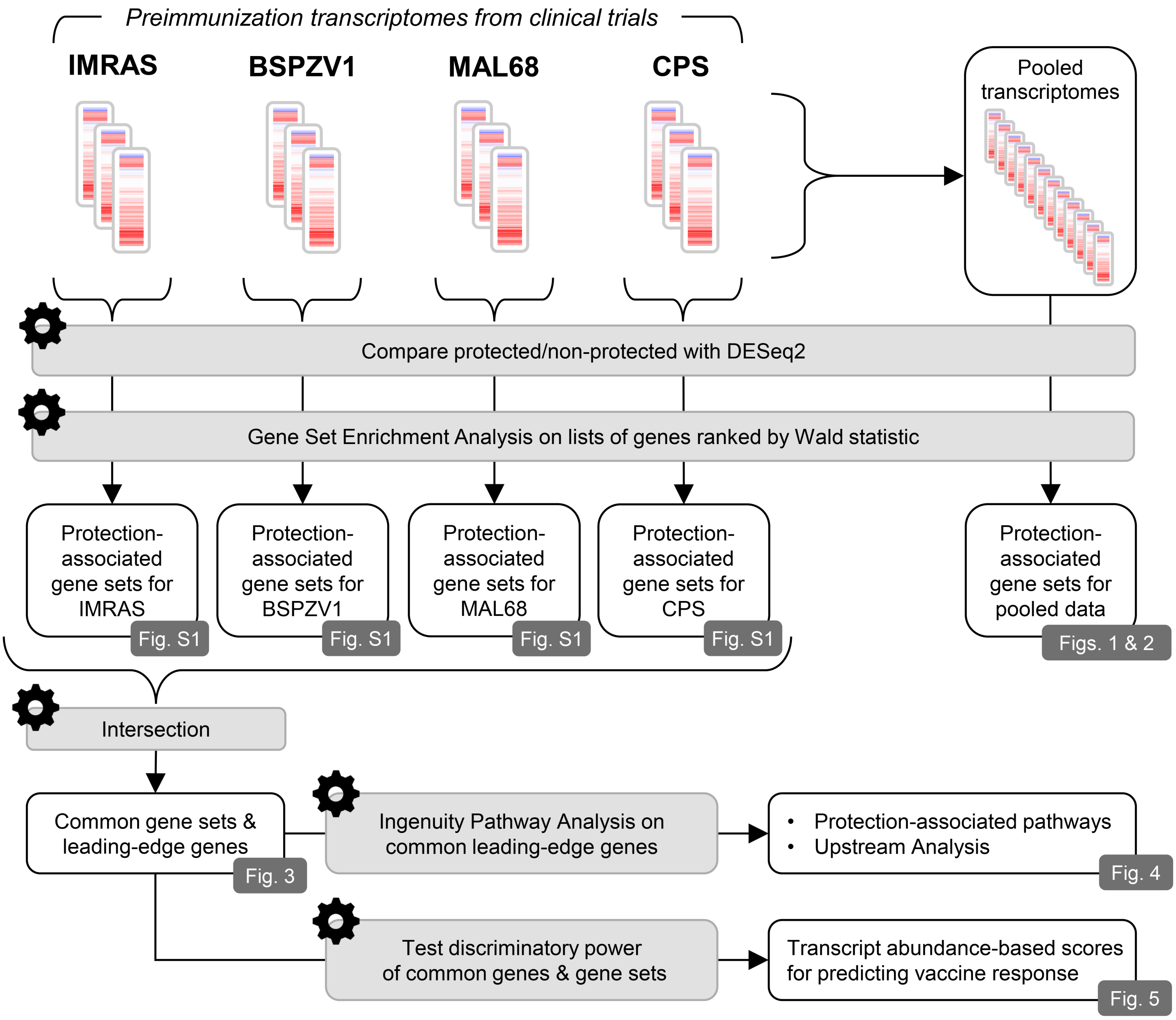

Supplementary Figure 2. Schematic showing the analysis pipeline used to identify preimmunization correlates of protection across four independent malaria vaccine trials. Gear icon indicates an analysis process as opposed to static data.

Supplementary Table 1. Leading-edge genes consistently appearing in gene sets commonly associated with protection across malaria vaccine trials. For each trial, the log_2 fold-change in transcript abundance between protected and non-protected subjects is shown. Mean, standard devation, maximum and minimum log_2 fold-changes across trials are also shown.

GeneName	PvsNP_Log2FoldChange_IMRAS	PvsNP_Log2FoldChange_BSPZV1	PvsNP_Log2FoldChange_MAL68	PvsNP_Log2FoldChange_CPS	Mean	StDev	Max	Min
ACSL1	0.383328766	0.580844354	0.151306458	1.037815123	0.538323675	0.376433023	1.037815123	0.151306458
AGTRAP	0.366413531	0.196757331	0.175946138	0.239137282	0.24456357	0.085382758	0.366413531	0.175946138
ALOX5	0.237557454	0.384406887	0.130425589	0.445906925	0.299574214	0.142673497	0.445906925	0.130425589
ATP6V0B	0.338852766	0.215246493	0.130090255	0.42602494	0.277553613	0.130931204	0.42602494	0.130090255
BCL3	0.26625933	0.425781964	0.075146723	1.012955704	0.44503593	0.404838521	1.012955704	0.075146723
BST1	0.314563152	0.341462649	0.121279517	0.329018058	0.276580844	0.10411607	0.341462649	0.121279517
BTG1	0.27051972	0.101553715	0.04913994	0.638637141	0.264962629	0.266424116	0.638637141	0.04913994
CAB39	0.192497533	0.148115569	0.033700354	0.179194283	0.138376935	0.072219856	0.192497533	0.033700354
CCRL2	0.248762816	0.159280628	0.132608539	0.420356702	0.240252171	0.129940519	0.420356702	0.132608539
CEBPD	0.50440913	0.158463384	0.331725972	0.414275888	0.352218593	0.147166681	0.50440913	0.158463384
CR1	0.254937312	0.521942275	0.110309239	0.339315279	0.306626026	0.171893066	0.521942275	0.110309239
CREB5	0.24044868	0.401120336	0.204653309	0.848407422	0.423657437	0.295775099	0.848407422	0.204653309
CXCL1	0.260424776	0.646867385	0.788914579	0.914359624	0.652641591	0.283392559	0.914359624	0.260424776
CXCR2	0.309717843	0.377810449	0.748689902	0.19504029	0.407814621	0.239438302	0.748689902	0.19504029
CYP4F3	0.725732403	0.435654104	0.863967927	0.489332506	0.628671735	0.201209922	0.863967927	0.435654104
DOK3	0.39750604	0.45725149	0.112054399	0.50692832	0.368435062	0.176677584	0.50692832	0.112054399
DYSF	0.215225347	0.566557144	0.180108388	0.615190048	0.394270232	0.228335069	0.615190048	0.180108388
ETV6	0.100816449	0.148051443	0.108627596	0.312024906	0.167380099	0.09862098	0.312024906	0.100816449
F5	0.319289671	0.327933954	0.081021001	0.602421308	0.332666483	0.213146624	0.602421308	0.081021001
FAM53C	0.358142764	0.318336758	0.137024482	0.313725184	0.281807297	0.098560168	0.358142764	0.137024482
FBXL5	0.188982217	0.151864501	0.110868635	0.372523157	0.206059627	0.115470316	0.372523157	0.110868635
FCAR	0.389206703	0.488496936	0.298274459	0.351606022	0.38189603	0.080264668	0.488496936	0.298274459
FKBP5	0.677251689	0.169206787	0.143987005	0.236465295	0.306727694	0.250080899	0.677251689	0.143987005
FPR1	0.476197047	0.365051263	0.220734963	0.378761703	0.360186244	0.105315213	0.476197047	0.220734963
FPR2	0.392353869	0.482814927	0.2486732	0.352827381	0.369167345	0.097020996	0.482814927	0.2486732
GCA	0.304923059	0.364066927	0.18807823	0.90902838	0.441524149	0.320132534	0.90902838	0.18807823
GNL2	-0.186886955	-0.152579362	-0.068112606	-0.572480919	-0.245014961	0.223943149	-0.068112606	-0.572480919
GNL3	-0.077800956	-0.073201111	-0.111447581	-0.418754259	-0.170300977	0.166510654	-0.073201111	-0.418754259
GOT1	-0.157488328	-0.199307723	-0.121531547	-0.736764468	-0.303773017	0.290405325	-0.121531547	-0.736764468
HAL	0.349108109	0.399935656	0.156662215	0.425383198	0.332772294	0.121613206	0.425383198	0.156662215
IGSF6	0.230480525	0.133266051	0.186067978	0.19832675	0.187035326	0.040443591	0.230480525	0.133266051
IL17RA	0.384355361	0.282544829	0.144013204	0.366973921	0.294471829	0.109720332	0.384355361	0.144013204
IL18RAP	0.347015617	0.488092046	0.202326154	0.642741413	0.420043808	0.188819914	0.642741413	0.202326154
IL1RN	0.208451104	0.251207369	0.253624601	0.551958223	0.316310324	0.158462864	0.551958223	0.208451104
IRAK3	0.499085004	0.391373489	0.148865204	0.606644036	0.411491934	0.195903411	0.606644036	0.148865204
ITGAM	0.262578314	0.250923776	0.066557673	0.37793466	0.239498605	0.128758579	0.37793466	0.066557673
KLHL2	0.460327602	0.232602556	0.106631463	0.377330385	0.294223001	0.156509041	0.460327602	0.106631463
LAGE3	-0.187670999	-0.084783729	-0.093068828	-0.855691474	-0.305303758	0.369881446	-0.084783729	-0.855691474
LAMP2	0.301194987	0.125149606	0.070177081	0.371856632	0.217094577	0.142668381	0.371856632	0.070177081
LAPTM5	0.225892621	0.172974456	0.045859825	0.407500492	0.213056849	0.150038362	0.407500492	0.045859825
LRP10	0.208246923	0.201744085	0.10096046	0.507973192	0.254731165	0.175826969	0.507973192	0.10096046
LRRK2	0.331892925	0.15506512	0.131074691	0.606091319	0.306031014	0.21916922	0.606091319	0.131074691
LYN	0.145219585	0.186083692	0.135354517	0.433984661	0.225160614	0.140937565	0.433984661	0.135354517
MANSC1	0.684780026	0.445944007	0.30181183	0.45756546	0.472525331	0.158246137	0.684780026	0.30181183
1-Mar	0.597478323	0.339709889	0.192890018	0.585331841	0.428852518	0.197099932	0.597478323	0.192890018
MEGF9	0.38031512	0.326949117	0.264718961	0.437359456	0.352335664	0.073785858	0.437359456	0.264718961
MGAM	0.501554737	0.421593002	0.382516437	0.673404879	0.494767263	0.12898603	0.673404879	0.382516437
MME	0.686501634	0.604198372	0.187190598	0.724898033	0.550697159	0.247513097	0.724898033	0.187190598
MOSPD2	0.179631437	0.240248543	0.195847196	0.48303118	0.274689589	0.141238073	0.48303118	0.179631437
MYD88	0.112044962	0.272203008	0.102497916	0.285770034	0.19312898	0.099370811	0.285770034	0.102497916
NCSTN	0.078988443	0.236104712	0.041697639	0.399292217	0.189020753	0.16354632	0.399292217	0.041697639
NDEL1	0.167711327	0.274116474	0.114215096	0.296077887	0.213030196	0.086499858	0.296077887	0.114215096
NFAM1	0.258971161	0.299462159	0.109475137	0.317164215	0.246268168	0.094392001	0.317164215	0.109475137
NFKBIZ	0.081148068	0.219666975	0.225786944	0.487910792	0.253628195	0.169868801	0.487910792	0.081148068
NPL	0.353223976	0.306466541	0.206913186	0.34578874	0.30309811	0.067325028	0.353223976	0.206913186
NSUN7	0.507471649	0.531330402	0.170595132	0.584966481	0.448590916	0.188142423	0.584966481	0.170595132
NUMB	0.213924886	0.286613139	0.109362318	0.26307761	0.218244488	0.078651694	0.286613139	0.109362318
OSM	0.3789314	0.635779254	0.225496901	0.845420102	0.521406914	0.274425209	0.845420102	0.225496901
PARP1	-0.236177249	-0.085705234	-0.038838592	-0.173601564	-0.13358066	0.088309774	-0.038838592	-0.236177249
PDK3	0.193463465	0.216895735	0.077021113	0.309014615	0.199098732	0.095451447	0.309014615	0.077021113
PFKFB3	0.270416034	0.577823756	0.094884432	0.323167217	0.31657286	0.199642788	0.577823756	0.094884432
PILRA	0.273220593	0.215104697	0.160213551	0.808872118	0.36435274	0.299916839	0.808872118	0.160213551
PLXDC2	0.328595759	0.282595519	0.098202516	0.614535777	0.330982393	0.213647668	0.614535777	0.098202516
PRKCD	0.255417452	0.266993535	0.046883058	0.172907139	0.185550296	0.101493634	0.266993535	0.046883058
PRR13	0.099199192	0.171480994	0.043201133	0.838121573	0.288000723	0.370487361	0.838121573	0.043201133
PTGS2	0.262343628	0.209033176	0.63263876	0.214556716	0.32964307	0.203410274	0.63263876	0.209033176
PTTG1IP	0.158319202	0.125654803	0.037469448	0.276543264	0.149496679	0.098889176	0.276543264	0.037469448
QPCT	0.499537261	0.509307792	0.138357426	0.86619936	0.50335046	0.297169576	0.86619936	0.138357426
REPS2	0.311668078	0.281611455	0.118764877	0.527201752	0.30981154	0.167884905	0.527201752	0.118764877
RNF149	0.275466207	0.243243312	0.065890711	0.545207931	0.28245204	0.197927254	0.545207931	0.065890711
RPL4	-0.060926732	-0.105179033	-0.093877003	-0.221848753	-0.12045788	0.070152341	-0.060926732	-0.221848753
RPL5	-0.082028615	-0.104176782	-0.06335589	-0.299667076	-0.137307091	0.109518449	-0.06335589	-0.299667076
RPL6	-0.123829916	-0.127680127	-0.048520766	-0.302540232	-0.15064276	0.107622728	-0.048520766	-0.302540232
RPS14	-0.151228249	-0.181059746	-0.087507863	-0.348496095	-0.192072988	0.111342771	-0.087507863	-0.348496095
RPS18	-0.22140867	-0.109525422	-0.107708645	-0.333322192	-0.192991232	0.107610393	-0.107708645	-0.333322192
RPS3	-0.063323019	-0.08369427	-0.073231826	-0.311777697	-0.133006703	0.119470549	-0.063323019	-0.311777697
RTN3	0.322718717	0.241142772	0.141312306	0.525949726	0.30778088	0.163271969	0.525949726	0.141312306
SELL	0.198781198	0.215640828	0.063507942	0.598641083	0.269142763	0.229976832	0.598641083	0.063507942
SHMT2	-0.078776141	-0.126952392	-0.02806222	-0.214440146	-0.112057725	0.079303047	-0.02806222	-0.214440146
SLC25A44	0.187179985	0.250441571	0.082864094	0.328295308	0.212195239	0.103753419	0.328295308	0.082864094
SLC2A3	0.399089901	0.4203967	0.136538983	0.572848556	0.382218535	0.181144935	0.572848556	0.136538983
SLC30A1	0.112755525	0.053007642	0.170117463	0.378744058	0.178656172	0.141702176	0.378744058	0.053007642
SNX27	0.253831545	0.226539319	0.168769728	0.577985108	0.306781425	0.184247254	0.577985108	0.168769728

SPI1	0.233534948	0.255118459	0.143510453	0.791843175	0.356001759	0.294553818	0.791843175	0.143510453
SSH2	0.218870573	0.264340358	0.086272211	0.339329409	0.227203138	0.10627391	0.339329409	0.086272211
STEAP4	0.561495919	0.283757894	0.305066907	0.663582526	0.453475812	0.18854049	0.663582526	0.283757894
TLR1	0.128221327	0.193728013	0.100787938	0.455430896	0.219542044	0.162020499	0.455430896	0.100787938
TLR4	0.350675799	0.333637129	0.163048857	0.532683904	0.345011422	0.151099079	0.532683904	0.163048857
TLR8	0.32083955	0.344551764	0.276192412	0.32073854	0.315580567	0.028548313	0.344551764	0.276192412
TM9SF2	0.238905201	0.072059343	0.052133303	0.248117955	0.152803951	0.105122874	0.248117955	0.052133303
TMEM154	0.245009688	0.179956958	0.092248868	0.283336403	0.200137979	0.083631696	0.283336403	0.092248868
TMEM167B	0.083791277	0.072299906	0.077678888	0.260364529	0.12353365	0.091341304	0.260364529	0.072299906
TOMM7	-0.2957149	-0.250304299	-0.123818256	-0.375988296	-0.261456438	0.105451139	-0.123818256	-0.375988296
TSHZ3	0.471662628	0.337230692	0.227175346	0.678468483	0.428634287	0.194258321	0.678468483	0.227175346
USP10	0.135906057	0.159492564	0.044748204	0.291171826	0.157829663	0.10173652	0.291171826	0.044748204
VNN2	0.264825377	0.418907914	0.20469039	0.582942269	0.367841487	0.16941736	0.582942269	0.20469039
WDFY3	0.182457309	0.400406559	0.14238355	0.453215143	0.29461564	0.155026473	0.453215143	0.14238355
YIPF1	0.157336855	0.094501512	0.158825976	0.564558231	0.243805643	0.215926176	0.564558231	0.094501512

Supplementary Table 2. Results from IPA Upstream Analysis on 98 leading-edge genes commonly associated with protection across malaria vaccine trials

Upstream Regulator	Expr Log Ratio	Molecule Type	p-value of overlap	B-H corrected p-value	Predicted Activation State	Activation z-score	Flags	Bias-corrected z-score	Target Molecules in Dataset
lipopolysaccharide		chemical drug	1.57E-14	3.94E-11	Activated	3.737	bias	1.795	ACSL1,ALOX5,BCL3,BTG1,CCRL2,CEBPD,CXCL1,CXCR2,ETV6,FPR1,FPR2,GCA,GNL3,IGSF6,IL17RA,IL18RAP,IL1RN,IRAK3,ITGAM,LRRK2,LYN,MME,MYD88,NFKBIZ,OSM,PFKFB3,PTGS2,RPS14,SELL,SLC2A3,SLC30A1,SPI1,STEAP4,TLR1,TLR4,TLR8,TMEM154
filgrastim		biologic drug	7.77E-12	9.72E-09	Activated	2.007		2.727	ACSL1,ALOX5,CR1,CXCL1,F5,FCAR,FKBP5,IL18RAP,IRAK3,ITGAM,MGAM,MME,PFKFB3,PILRA,PTGS2,TLR4,TLR8
dexamethasone		chemical drug	5.93E-11	4.04E-08		1.251		0.699	ACSL1,ALOX5,BCL3,BTG1,CEBPD,CR1,CXCL1,F5,FCAR,FKBP5,FPR2,GOT1,IL18RAP,IL1RN,IRAK3,ITGAM,LAGE3,LAPTM5,LRRK2,LYN,MEGF9,MGAM,MME,NFKBIZ,OSM,PFKFB3,PILRA,PTGS2,RPL5,SELL,SLC2A3,SPI1,TLR4,TLR8
TNF		cytokine	6.46E-11	4.04E-08	Activated	3.658	bias	2.385	ACSL1,ALOX5,BCL3,BTG1,CEBPD,CXCL1,CXCR2,ETV6,FCAR,FPR1,FPR2,IL1RN,IRAK3,ITGAM,LYN,MYD88,NFKBIZ,NUMB,OSM,PARP1,PDK3,PLXDC2,PRKCD,PTGS2,RNF149,RPS3,SELL,STEAP4,TLR1,TLR4,TLR8
salmonella minnesota R595 lipopolysaccharides		chemical - endogenous non-mammalian	1.33E-10	6.63E-08	Activated	3.13	bias		ACSL1,BCL3,CCRL2,CEBPD,CXCL1,FPR1,FPR2,NFKBIZ,PILRA,PTGS2
IL10		cytokine	1.82E-10	7.57E-08		0.831		0.587	BCL3,CR1,FKBP5,FPR1,IL17RA,IL1RN,ITGAM,LAMP2,LYN,MYD88,OSM,PTGS2,SELL,TLR1,TLR4,TLR8
resiquimod		chemical drug	4.09E-10	1.46E-07		1.97	bias	0.705	CCRL2,CREB5,CXCL1,IL18RAP,IRAK3,LYN,MYD88,NFKBIZ,PTGS2,SPI1,TLR4,TLR8,TMEM154
CSF2		cytokine	1.38E-09	4.32E-07	Activated	3.645	bias		ALOX5,BCL3,CCRL2,CXCL1,FPR2,IL1RN,IRAK3,ITGAM,LAMP2,MME,OSM,PTGS2,SLC2A3,SPI1,TLR1,TLR4
Immunoglobulin		complex	1.70E-09	4.73E-07		-1.186		-1.419	ACSL1,BCL3,CEBPD,CXCL1,CXCR2,DYSF,FKBP5,FPR1,HAL,IL1RN,ITGAM,NFKBIZ,OSM,PDK3,PTGS2,RPS3,SELL,SPI1,TLR8
MYD88	0.193	other	3.77E-09	9.44E-07	Activated	2.93	bias		ACSL1,BCL3,CCRL2,CEBPD,CXCL1,FPR1,FPR2,IL1RN,NFKBIZ,PARP1,PILRA,PTGS2
TICAM1		other	1.55E-08	3.24E-06	Activated	3	bias		ACSL1,BCL3,CCRL2,CEBPD,FPR1,FPR2,NFKBIZ,PILRA,PTGS2
IFNG		cytokine	1.48E-08	3.24E-06	Activated	4.385	bias	3.025	BCL3,BST1,BTG1,CCRL2,CEBPD,CXCL1,FKBP5,FPR2,IL17RA,IL18RAP,IL1RN,ITGAM,LYN,MYD88,NFKBIZ,OSM,PFKFB3,PRKCD,PTGS2,SELL,SPI1,TLR1,TLR4,TLR8
CAMP		other	1.93E-08	3.72E-06		1.924	bias	0.829	CXCL1,CXCR2,FPR2,IL1RN,ITGAM,MYD88,PTGS2,TLR4
IL3		cytokine	3.32E-08	5.94E-06		1.029	bias		ITGAM,LYN,OSM,RPL4,RPL5,RPL6,RPS14,RPS3,SELL,SLC2A3,SPI1,TLR4
CSF3		cytokine	8.64E-08	1.42E-05		1.995		1.413	CEBPD,CXCR2,FPR1,IL1RN,ITGAM,SELL,SPI1,TLR4,TLR8
5-O-mycolyl-beta-araf-(1->2)-5-O-mycolyl-alpha-araf-(1->1')-glycerol		chemical - endogenous non-mammalian	9.05E-08	1.42E-05		1.89	bias		ACSL1,BCL3,CXCL1,CXCR2,IL18RAP,NFKBIZ,PTGS2
TCL1A		transcription regulator	1.25E-07	1.83E-05					CXCR2,FKBP5,FPR1,IGSF6,IL17RA,ITGAM,STEAP4
IL17A		cytokine	3.15E-07	4.38E-05	Activated	3.048	bias	1.526	BCL3,CEBPD,CXCL1,IL17RA,IL1RN,LRRK2,NFKBIZ,PTGS2,STEAP4,TLR4
MYC		transcription regulator	3.68E-07	4.85E-05		-1.798	bias	-3.026	ALOX5,ATP6V0B,CEBPD,FKBP5,GOT1,ITGAM,LAMP2,MYD88,NDEL1,PARP1,PFKFB3,PTGS2,RPL4,RPL5,RPL6,RPS14,RPS18,RPS3,SHMT2,SLC2A3
tretinoin		chemical - endogenous mammalian	5.03E-07	6.29E-05	Activated	3.276	bias	2.175	ACSL1,ALOX5,BCL3,BTG1,CCRL2,CXCL1,CYP4F3,ETV6,FCAR,GCA,ITGAM,LAMP2,LRRK2,LYN,NUMB,PRKCD,PTGS2,RPL4,RPL5,RPL6,RPS14,RPS3,SELL,SPI1
peptidoglycan		chemical - endogenous non-mammalian	5.68E-07	6.77E-05		0.999	bias		CXCL1,FPR2,IL1RN,MYD88,PTGS2,SELL,TLR4
IL1B		cytokine	6.61E-07	7.46E-05	Activated	3.669	bias	2.597	BCL3,CCRL2,CEBPD,CXCL1,FKBP5,FPR2,IL18RAP,IL1RN,IRAK3,ITGAM,MYD88,NFKBIZ,OSM,PLXDC2,PRKCD,PTGS2,TLR4,TLR8
calcitriol		chemical drug	6.86E-07	7.46E-05		0.079		0.272	ACSL1,ALOX5,BCL3,CEBPD,CXCL1,IL1RN,ITGAM,MYD88,PDK3,PRKCD,PTGS2,TLR4,TLR8,TSHZ3
Interferon alpha		group	7.62E-07	7.52E-05	Activated	2.754	bias	1.273	BCL3,CXCL1,IL17RA,IL18RAP,IL1RN,ITGAM,MYD88,PTGS2,SLC2A3,TLR1,TLR4,TLR8
FAS		transmembrane receptor	7.82E-07	7.52E-05					CCRL2,CEBPD,CXCL1,CXCR2,F5,FCAR,FPR1,IL17RA,MME,OSM,TLR8
methylprednisolone		chemical drug	7.26E-07	7.52E-05		-0.749		-0.809	ACSL1,BTG1,CYP4F3,GNL3,GOT1,HAL,IGSF6,IL1RN,IRAK3,LYN,SLC2A3,SLC30A1,TLR1,TLR4
NEIL2		enzyme	1.09E-06	1.01E-04					CXCR2,IL1RN,MYD88,PTGS2,TLR1,TLR4
torin1		chemical reagent	1.40E-06	1.25E-04					ATP6V0B,PTGS2,RPL4,RPL5,RPL6,RPS14,RPS18,RPS3
ANXA1		enzyme	1.52E-06	1.31E-04		-0.037	bias	0.498	FPR2,ITGAM,PTGS2,SELL,TLR4
LARP1		translation regulator	1.70E-06	1.42E-04	Activated	2.449	bias		RPL4,RPL5,RPL6,RPS14,RPS18,RPS3
IL18		cytokine	2.09E-06	1.69E-04	Activated	2.573	bias	1.299	CCRL2,IL18RAP,ITGAM,NFKBIZ,PTGS2,SELL,SLC2A3,TLR4
lemairamin		chemical reagent	2.78E-06	2.18E-04					MYD88,PTGS2,TLR4
BCR-ABL1		fusion gene/product	2.92E-06	2.21E-04		0.762	bias	0.003	MME,NUMB,OSM,PDK3,PRKCD,SLC2A3
ethanol		chemical - endogenous mammalian	3.49E-06	2.57E-04	Activated	2.968		2.291	ALOX5,CEBPD,IL1RN,ITGAM,MME,MYD88,PARP1,PTGS2,TLR1,TLR4,TLR8
IL33		cytokine	4.16E-06	2.97E-04	Activated	2.969	bias	1.523	BCL3,CXCR2,FPR1,FPR2,IL1RN,IRAK3,ITGAM,NFKBIZ,PILRA,SPI1
mannan		chemical - endogenous mammalian	4.44E-06	3.08E-04					IRAK3,PTGS2,TLR4
TCR		complex	5.80E-06	3.72E-04		0.192	bias	-0.91	BCL3,F5,MYD88,RPL4,RPL5,RPL6,RPS3,SELL,SLC2A3,TLR4
JAK2		kinase	5.55E-06	3.72E-04		1.057	bias		ALOX5,F5,ITGAM,LYN,NFKBIZ,OSM,PTGS2

TGFB1		growth factor	5.77E-06	3.72E-04		0.694		-0.162	ALOX5,BCL3,BTG1,CAB39,CCRL2,CXCL1,CXCR2,F5,FCAR,FPR2,IL1RN,IRAK3,ITGAM,LAMP2,MYD88,OSM,PILRA,PTGS2,SHMT2,SLC2A3,SPI1,TLR4,WDFY3
cycloheximide		chemical reagent	6.98E-06	4.37E-04		0.147		0.448	CEBPD,CXCL1,GOT1,IL1RN,ITGAM,MME,OSM,PFKFB3,PTGS2,SLC30A1
miR-155-5p (miRNAs w/seed UAAUGCU)		mature microRNA	8.34E-06	5.09E-04	Inhibited	-2.578	bias		CXCL1,MOSPD2,MTARC1,MYD88,PTGS2,SLC30A1,SPI1
TLR4	0.345	transmembrane receptor	9.33E-06	5.43E-04	Activated	2.076	bias		CCRL2,CEBPD,CXCR2,IRAK3,ITGAM,LRRK2,MYD88,NFKBIZ,PTGS2,TLR4
cholesterol		chemical - endogenous mammalian	9.20E-06	5.43E-04		1.255		0.579	ACSL1,CR1,IL1RN,ITGAM,LAPTM5,MME,PTGS2,TLR4
Pam3-Cys-Ser-Lys4		chemical reagent	1.18E-05	6.69E-04		1.673	bias		CXCR2,MYD88,NFKBIZ,PTGS2,SPI1,TLR1,TLR4
IL4		cytokine	1.20E-05	6.70E-04		1.573	bias	0.056	ACSL1,ALOX5,BCL3,CXCL1,FKBP5,FPR1,FPR2,IL18RAP,IL1RN,LAMP2,NFKBIZ,OSM,PRKCD,PTGS2,SELL,TLR4,USP10
tyrphostin AG 1296		chemical - kinase inhibitor	1.30E-05	7.05E-04					FCAR,ITGAM,PTGS2
OSCAR		other	1.43E-05	7.38E-04		1	bias	0.332	CXCL1,IL1RN,ITGAM,TLR8
PARP1	-0.134	enzyme	1.45E-05	7.38E-04	Activated	2.219	bias	1.428	CXCL1,CXCR2,PARP1,PTGS2,TLR1,TLR4
camptothecin		chemical drug	1.40E-05	7.38E-04		1.342		0.836	CCRL2,CXCL1,CXCR2,F5,FAM53C,FCAR,FPR1,IL17RA,OSM,PTGS2,SLC30A1,WDFY3
Alpha catenin		group	1.52E-05	7.58E-04	Inhibited	-2.449	bias		BCL3,ITGAM,LYN,NFKBIZ,PTGS2,STEAP4
E. coli B5 lipopolysaccharide		chemical - endogenous non-mammalian	1.91E-05	9.17E-04	Activated	2.76	bias	1.778	BCL3,CXCR2,IL17RA,IL18RAP,IRAK3,MYD88,PTGS2,TLR4
epoxyeicosatrienoic acid		chemical - other	1.88E-05	9.17E-04					ALOX5,PTGS2
IL13		cytokine	2.22E-05	1.05E-03		1.577	bias	0.611	ACSL1,BCL3,CXCL1,CXCR2,FPR2,IL1RN,IRAK3,PTGS2,TLR1,TLR4
IL1		group	2.49E-05	1.13E-03	Activated	2.034	bias	0.703	CEBPD,CXCL1,FPR1,IL1RN,ITGAM,PTGS2,SELL,SLC2A3,TLR4
TLR2		transmembrane receptor	2.45E-05	1.13E-03	Activated	2.379	bias	1.399	CEBPD,FPR2,IL1RN,IRAK3,MYD88,PTGS2,TLR4
TP53		transcription regulator	2.69E-05	1.20E-03		-1.748		-2.105	ACSL1,ALOX5,BCL3,BTG1,CEBPD,CXCL1,CXCR2,F5,FKBP5,GNL3,IL17RA,IL1RN,LAMP2,NFAM1,PARP1,PDK3,PFKFB3,PTGS2,RPL5,RPS18,RPS3,TLR1
naringenin		chemical - endogenous non-mammalian	3.36E-05	1.47E-03					CXCL1,LYN,PTGS2,TLR4
CXCL8		cytokine	3.55E-05	1.48E-03		0.387	bias	-0.368	CR1,CXCR2,ITGAM,PTGS2,SELL
EDNRA		transmembrane receptor	3.53E-05	1.48E-03					ITGAM,PTGS2,SELL
mycophenolic acid		chemical drug	3.55E-05	1.48E-03	Activated	2.236	bias	1.187	GNL3,MGAM,MME,PTGS2,SLC30A1
Collagen type II		complex	4.04E-05	1.61E-03		0		-0.113	CXCL1,CXCR2,IL1RN,TLR8
YAP1		transcription regulator	3.94E-05	1.61E-03					PTGS2,RPL4,RPL5,RPL6,RPS14,RPS18,RPS3,SLC2A3
ELANE		peptidase	4.04E-05	1.61E-03					CEBPD,OSM,SPI1,TLR4
TGFBR2		kinase	4.32E-05	1.69E-03		-1.134	bias	-0.14	ALOX5,BCL3,FKBP5,FPR1,ITGAM,NFKBIZ,PTGS2,SLC2A3,WDFY3
IL6		cytokine	4.43E-05	1.71E-03	Activated	2.493	bias	1.339	BCL3,CEBPD,CXCL1,FPR2,IL1RN,ITGAM,MYD88,PTGS2,SPI1,STEAP4,TLR1,TLR4,TLR8
SP1		transcription regulator	4.55E-05	1.72E-03		1.185	bias		ACSL1,ALOX5,CEBPD,FPR2,IRAK3,ITGAM,PARP1,PRKCD,PTGS2,SELL,SLC2A3
MLXIPL		transcription regulator	4.72E-05	1.76E-03	Inhibited	-2.449	bias		RPL4,RPL5,RPL6,RPS14,RPS18,RPS3
EPO		cytokine	4.89E-05	1.76E-03		0.225	bias	-0.458	BCL3,BTG1,FPR1,LAPTM5,OSM,PRKCD,PTGS2,TLR4
Pam3-Cys		chemical toxicant	4.83E-05	1.76E-03					FPR1,IL1RN,MYD88,TLR4
poly rI:rC-RNA		biologic drug	4.93E-05	1.76E-03	Activated	3.066	bias		CEBPD,CXCL1,FPR1,ITGAM,LYN,MYD88,NFKBIZ,PRKCD,PTGS2,TLR1,TLR4,YIPF1
zoledronic acid		chemical drug	5.26E-05	1.83E-03		-0.927		-0.486	IRAK3,MYD88,PTGS2,TLR4
ZFTA-RELA		fusion gene/product	5.26E-05	1.83E-03	Activated	2	bias		BCL3,IL1RN,PRKCD,PTGS2
CNPY3		other	5.64E-05	1.93E-03					TLR1,TLR4
N-formyl-Met-Leu-Phe		chemical reagent	5.72E-05	1.93E-03		1.071	bias	0.265	ITGAM,OSM,PTGS2,SELL
budesonide		chemical drug	6.16E-05	2.04E-03	Inhibited	-2.121	bias	-1.29	CAB39,CXCR2,CYP4F3,IL1RN,ITGAM,LAPTM5,MEGF9,PFKFB3
Cyp2c23		enzyme	6.27E-05	2.04E-03					F5,ITGAM,PLXDC2
HOXA3		transcription regulator	6.21E-05	2.04E-03		-1.332		-1.067	ALOX5,IL17RA,PRKCD,SPI1
JUN		transcription regulator	6.86E-05	2.15E-03		1.121	bias	0.132	BCL3,BTG1,CXCL1,GOT1,IRAK3,ITGAM,LAMP2,NFKBIZ,OSM,PTGS2
CEBPB		transcription regulator	7.05E-05	2.15E-03		-0.088	bias		CCRL2,CEBPD,FCAR,IL1RN,IRAK3,LYN,NFKBIZ,PRKCD,PTGS2,TLR4,TLR8
CRP		other	6.72E-05	2.15E-03		0.807	bias	0.081	FCAR,IL1RN,ITGAM,SELL
1-methyl-4-phenyl-1,2,3,6-tetrahydropyridine		chemical toxicant	7.03E-05	2.15E-03		1.953	bias	1.393	FKBP5,ITGAM,OSM,PTGS2,TLR4
erlotinib		chemical drug	7.03E-05	2.15E-03		0.156	bias	-0.717	CXCL1,MME,MYD88,SLC2A3,TLR4
NR1H3		ligand-dependent nuclear receptor	7.33E-05	2.21E-03		-1.969		-2.127	FPR1,FPR2,IL1RN,ITGAM,PTGS2,RPS14,TLR1
prostaglandin E2		chemical - endogenous mammalian	8.40E-05	2.50E-03		1.889	bias	1.245	BTG1,CEBPD,CXCL1,IL1RN,ITGAM,OSM,PTGS2,SELL
CEBPD	0.352	transcription regulator	8.50E-05	2.50E-03					CEBPD,CXCL1,ITGAM,PTGS2,TLR8
IL1RL1		transmembrane receptor	8.70E-05	2.53E-03					ITGAM,TLR1,TLR4
1,25-dihydroxyvitamin D		chemical drug	9.11E-05	2.62E-03		-0.762		-0.87	SPI1,TLR1,TLR4,TLR8
Fibrinogen		complex	1.01E-04	2.88E-03					BCL3,CXCL1,ITGAM
ERK		group	1.06E-04	2.93E-03		1.08	bias	-0.129	CEBPD,CXCL1,GOT1,ITGAM,NFKBIZ,PTGS2,SELL
CEBPE		transcription regulator	1.05E-04	2.93E-03					IL1RN,ITGAM,PTGS2,SELL
IL32		cytokine	1.13E-04	3.10E-03		-1.044	bias	-1.898	CXCL1,ITGAM,PTGS2,SPI1
VIP		other	1.16E-04	3.17E-03		-1.27		-1.081	CEBPD,PTGS2,TLR1,TLR4,TLR8
APOA1		transporter	1.21E-04	3.25E-03		-0.882	bias	-0.364	ITGAM,MYD88,PTGS2,TLR4
NFkB (complex)		complex	1.29E-04	3.43E-03	Activated	2.741	bias		BCL3,CEBPD,CXCL1,FPR2,IL1RN,ITGAM,LAMP2,NFKBIZ,PRKCD,PTGS2,TLR4
KITLG		growth factor	1.38E-04	3.64E-03	Activated	2.148	bias		ALOX5,BTG1,IL1RN,LAPTM5,MME,OSM,PTGS2
hyaluronic acid		chemical - endogenous mammalian	1.44E-04	3.71E-03					BCL3,IRAK3,MYD88,PTGS2,TLR4
CpG oligonucleotide		chemical drug	1.44E-04	3.71E-03		1.987	bias		LYN,MYD88,NFKBIZ,PRKCD,PTGS2,SLC2A3
vasoactive intestinal peptide		biologic drug	1.52E-04	3.85E-03					IL17RA,MYD88,TLR4

resolvin D2		chemical - endogenous mammalian	1.52E-04	3.85E-03					MYD88,SELL,TLR4
ATP		chemical - endogenous mammalian	1.69E-04	4.20E-03		0.129	bias	-0.551	IL1RN,ITGAM,PTGS2,SELL,TLR4
CEBPA		transcription regulator	1.72E-04	4.20E-03	Activated	2.615		1.985	ACSL1,BTG1,CEBPD,FCAR,IL1RN,ITGAM,PTGS2,SPI1,STEAP4
HSD17B4		enzyme	1.73E-04	4.20E-03					ACSL1,ALOX5,PTGS2
EIF4EBP2		translation regulator	1.73E-04	4.20E-03					CEBPD,IL1RN,PTGS2
DUSP1		phosphatase	1.76E-04	4.23E-03	Inhibited	-2.163		-2.026	CXCL1,IL1RN,OSM,PARP1,PTGS2
IL2		cytokine	1.81E-04	4.31E-03		1.255	bias	-0.01	FPR2,GNL3,IL18RAP,IL1RN,LAPTM5,MME,OSM,PTGS2,SELL,SLC2A3,SLC30A1
TNFSF11		cytokine	1.83E-04	4.31E-03		0.943	bias		BST1,FPR1,IL1RN,ITGAM,NFKBIZ,PTGS2,TLR4
IntegrinÎ±		group	1.87E-04	4.33E-03					ITGAM,TLR4
Ho		group	1.87E-04	4.33E-03					CXCR2,ITGAM
AHI1		other	1.95E-04	4.47E-03					IL1RN,LAPTM5,REPS2
LDL		complex	2.00E-04	4.55E-03		1.569	bias	0.535	CXCL1,IL17RA,IL1RN,IRAK3,PARP1,PTGS2,TLR4
ECSIT		transcription regulator	2.18E-04	4.88E-03					BCL3,IL1RN,PTGS2
AS1842856		chemical reagent	2.18E-04	4.88E-03					ALOX5,CXCL1,PTGS2
PD98059		chemical - kinase inhibitor	2.29E-04	5.07E-03		-1.899	bias	-0.497	CEBPD,CXCL1,FKBP5,FPR2,IL1RN,ITGAM,LAMP2,OSM,PTGS2,SELL
RICTOR		other	2.33E-04	5.12E-03		1.912	bias	3.147	ATP6V0B,PFKFB3,PTGS2,RPL4,RPL6,RPS18,RPS3
TREM1		transmembrane receptor	2.40E-04	5.22E-03		0.588	bias	-0.328	CCRL2,CXCL1,MYD88,PTGS2,PTTG1IP,TLR4
MALP-2s		chemical reagent	2.44E-04	5.26E-03					ITGAM,PTGS2,TLR4
azoxymethane		chemical toxicant	2.50E-04	5.36E-03					CXCR2,PARP1,PRKCD,PTGS2
E. coli B4 lipopolysaccharide		chemical toxicant	2.54E-04	5.38E-03	Activated	2.509	bias	1.315	F5,FPR1,FPR2,IL1RN,IRAK3,PTGS2,TLR4
mir-155		microRNA	2.56E-04	5.38E-03		-0.879	bias	-0.098	IRAK3,MME,MYD88,PTGS2,SPI1
lipoteichoic acid		chemical - endogenous non-mammalian	2.65E-04	5.52E-03		1.997	bias		IL1RN,MYD88,PTGS2,TLR4
SND1		enzyme	2.71E-04	5.56E-03					LAPTM5,PTGS2,QPCT
NPC2		transporter	2.71E-04	5.56E-03					CREB5,PTGS2,TLR4
ABCD2		transporter	2.79E-04	5.68E-03					ALOX5,PTGS2
COP1		enzyme	3.00E-04	6.06E-03					CXCR2,FPR1,FPR2
E. coli lipopolysaccharide		chemical - endogenous non-mammalian	3.10E-04	6.20E-03		1.96	bias		CEBPD,CXCL1,PTGS2,TLR4
IL12 (complex)		complex	3.12E-04	6.20E-03	Activated	2.177	bias	1.139	BCL3,IL18RAP,MYD88,PTGS2,SELL,TLR4
IDR-1002		chemical reagent	3.31E-04	6.53E-03					CXCL1,CXCR2,TLR4
SPI1	0.356	transcription regulator	3.40E-04	6.64E-03	Activated	2.57	bias	1.758	IL1RN,IRAK3,ITGAM,MME,PTGS2,SPI1,TLR4
glucocorticoid		chemical drug	3.75E-04	7.17E-03		-1.253		-1.176	ALOX5,BCL3,CEBPD,CXCL1,GOT1,IL1RN,PTGS2
PGR		ligand-dependent nuclear receptor	3.75E-04	7.17E-03		0.686	bias	-0.284	ACSL1,BTG1,CEBPD,FKBP5,GOT1,PFKFB3,PTGS2
CBFA2T3		transcription regulator	3.73E-04	7.17E-03		-1.213	bias	-0.662	BTG1,CYP4F3,ITGAM,LYN,PFKFB3
TCIM		other	3.90E-04	7.34E-03					CXCL1,PTGS2
KLF12		transcription regulator	3.90E-04	7.34E-03					IL1RN,OSM
zymosan		chemical - endogenous non-mammalian	3.98E-04	7.44E-03					CR1,FPR2,ITGAM,PTGS2
ZBTB16		transcription regulator	4.10E-04	7.54E-03		0		0.128	GOT1,IL18RAP,ITGAM,NFKBIZ,PTGS2,SELL
CTNNB1		transcription regulator	4.10E-04	7.54E-03					CCRL2,CXCL1,MME,NUMB,PTGS2,QPCT,RPL4,RPL5,RPL6,RPS14,RPS18,RPS3,SPI1
IgG		complex	4.38E-04	7.95E-03		-0.476		-0.084	BCL3,CEBPD,IL1RN,ITGAM,PTGS2,SLC2A3,TLR4
IL10RA		transmembrane receptor	4.38E-04	7.95E-03		-0.378		-0.441	ACSL1,ALOX5,IL1RN,ITGAM,NPL,REPS2,SELL
IL27		cytokine	4.67E-04	8.41E-03		1	bias	0.243	CEBPD,CR1,IL1RN,ITGAM,PTGS2
IL12 (family)		group	4.97E-04	8.81E-03	Activated	2.2	bias	1.339	BCL3,IL18RAP,NFKBIZ,SELL,SLC2A3
TGM2		enzyme	4.95E-04	8.81E-03	Activated	2.449	bias	1.651	CCRL2,CYP4F3,GCA,ITGAM,LRRK2,SELL
STAT3		transcription regulator	5.08E-04	8.95E-03	Activated	2.429	bias	1.354	BCL3,CCRL2,CEBPD,CXCR2,IL1RN,ITGAM,MYD88,NFKBIZ,PTGS2,SPI1
amoxicillin		chemical drug	5.19E-04	9.07E-03					TLR4,TLR8
NLRP12		other	5.62E-04	9.76E-03					BCL3,IL1RN,TLR4
cytokine		group	5.94E-04	1.03E-02	Activated	2.142	bias		ALOX5,BCL3,CXCL1,PTGS2,TLR4
CERK		kinase	6.08E-04	1.03E-02					CCRL2,CXCL1,MYD88
NQO1		enzyme	6.08E-04	1.03E-02					FPR1,PTGS2,SPI1
mifepristone		chemical drug	6.01E-04	1.03E-02	Inhibited	-2.589		-2.175	CEBPD,FKBP5,ITGAM,MME,NPL,PFKFB3,PTGS2
Nr1h		group	6.30E-04	1.05E-02		-1.213		-0.73	BCL3,CXCL1,NFKBIZ,PTGS2,TLR4
niacinamide		chemical - endogenous mammalian	6.26E-04	1.05E-02		1.98		2.192	AGTRAP,ITGAM,PARP1,PTGS2
ABCD1		transporter	6.65E-04	1.05E-02					ALOX5,PTGS2
ALOX5	0.3	enzyme	6.57E-04	1.05E-02					ALOX5,MYD88,PTGS2
CTSK		peptidase	6.65E-04	1.05E-02					PTGS2,TLR4
diclofenac		chemical drug	6.53E-04	1.05E-02		-0.415	bias	-1.181	ITGAM,PTGS2,SELL,SHMT2
flufenamic acid		chemical drug	6.65E-04	1.05E-02					PTGS2,SELL
actinomycin D		biologic drug	6.48E-04	1.05E-02	Inhibited	-2.37	bias	-1.503	CXCL1,FPR1,OSM,PFKFB3,PTGS2,SLC30A1
tyrphostin AG 127		chemical - kinase inhibitor	6.65E-04	1.05E-02					PARP1,PTGS2
L-N6-(1-iminoethyl)-lysine		chemical reagent	6.65E-04	1.05E-02					ITGAM,PTGS2
EIF4EBP1		translation regulator	7.08E-04	1.11E-02					CEBPD,IL1RN,PTGS2
tetradecanoylphorbol acetate		chemical drug	7.27E-04	1.14E-02		1.75	bias	0.466	BCL3,CXCR2,FKBP5,IL1RN,IRAK3,ITGAM,MME,OSM,PRKCD,PTGS2,SELL,TLR1,TLR4,TLR8
TLR9		transmembrane receptor	7.38E-04	1.14E-02		1.446	bias		LAMP2,MYD88,NFKBIZ,PFKFB3,PTGS2,TLR4
indomethacin		chemical drug	7.38E-04	1.14E-02		0.941		0.492	ACSL1,ALOX5,ATP6V0B,ITGAM,PTGS2,SELL
cardiotoxin		chemical - other	7.69E-04	1.15E-02		1.982	bias		CXCR2,FPR1,IL1RN,ITGAM,OSM,TSHZ3

Mek		group	7.67E-04	1.15E-02					GNL3,MANSC1,MME,OSM,PTGS2,PTTG1IP,STEAP4
ADIPOQ		other	7.69E-04	1.15E-02		1.073		0.831	ACSL1,BCL3,CXCL1,PTGS2,SLC2A3,SPI1
TLR7/8		group	7.61E-04	1.15E-02					NFKBIZ,PTGS2,REPS2
N-acetyl-L-cysteine		chemical drug	7.53E-04	1.15E-02		0.084	bias	1.125	GNL3,ITGAM,NFKBIZ,PARP1,PTGS2,TLR4
ADCY7		enzyme	8.29E-04	1.22E-02					TLR1,TLR4
IL9R		transmembrane receptor	8.29E-04	1.22E-02					BCL3,SELL
SATB1		transcription regulator	8.32E-04	1.22E-02		-0.549		-0.274	F5,IL18RAP,PTGS2,SELL,SPI1
IKZF1		transcription regulator	8.36E-04	1.22E-02		-1.671	bias	-0.665	LYN,NFKBIZ,SLC2A3,SPI1,TLR4,WDFY3
progesterone		chemical - endogenous mammalian	8.63E-04	1.26E-02		1.597		1.029	ACSL1,CEBPD,FKBP5,MME,MYD88,NPL,PDK3,PFKFB3,PTGS2,SLC2A3
Salmonella enterica serotype abortus equi lipopolysaccharide		chemical toxicant	8.78E-04	1.26E-02	Activated	2.213	bias		CCRL2,CXCL1,NFKBIZ,PTGS2,PTTG1IP
SELPLG		other	8.76E-04	1.26E-02					BST1,ITGAM,PRKCD
lysophosphatidic acid		chemical - other	9.34E-04	1.33E-02		1.943	bias		CXCL1,FKBP5,ITGAM,PTGS2
CpG ODN 2006		chemical reagent	9.34E-04	1.33E-02		-0.069	bias		MYD88,NFKBIZ,SELL,TLR4
NFKBIA		transcription regulator	9.42E-04	1.33E-02		1.121		0.666	BCL3,CEBPD,CXCL1,IL1RN,PTGS2,RPS18,TLR4,USP10
DIO3		enzyme	9.75E-04	1.37E-02	Inhibited	-2.236		-2.015	ALOX5,F5,ITGAM,LAPTM5,NFAM1
Collagen(s)		complex	1.00E-03	1.39E-02					BCL3,ITGAM,LAMP2
U73122		chemical reagent	1.00E-03	1.39E-02					ITGAM,PTGS2,TLR4
phospholipid		chemical - endogenous mammalian	1.01E-03	1.40E-02					ALOX5,PTGS2
KLF3		transcription regulator	1.02E-03	1.40E-02		-0.555		-0.047	BCL3,DOK3,IL17RA,IL1RN,LRRK2,OSM,SELL
TLR3		transmembrane receptor	1.04E-03	1.42E-02		1.176	bias		IL1RN,MYD88,NFKBIZ,PFKFB3,PTGS2,TLR4
Hbb-b2		other	1.07E-03	1.45E-02					CXCR2,ITGAM,SPI1
IL21		cytokine	1.08E-03	1.46E-02		1.98	bias	1.004	CCRL2,IL18RAP,MYD88,SELL,TLR4
FOXO1		transcription regulator	1.11E-03	1.49E-02		0.878	bias	-0.003	ALOX5,ATP6V0B,HAL,IL17RA,ITGAM,LAMP2,PRKCD,SELL
RELA		transcription regulator	1.11E-03	1.49E-02	Activated	2.38	bias		BCL3,CXCL1,IL1RN,LYN,PARP1,PRKCD,PTGS2,SPI1
mir-146		microRNA	1.14E-03	1.51E-02					MYD88,PTGS2,TLR4
PPRC1		transcription regulator	1.21E-03	1.58E-02					NFKBIZ,PTGS2,TMEM154
ABCA1		transporter	1.21E-03	1.58E-02					MYD88,PTGS2,TLR4
CACNA1A		ion channel	1.21E-03	1.58E-02					BTG1,ITGAM
sirolimus		chemical drug	1.23E-03	1.60E-02		1	bias	1.875	ATP6V0B,BCL3,FPR1,RPL5,RPS14,RPS18,RPS3,SELL,SLC2A3
SB203580		chemical - kinase inhibitor	1.25E-03	1.62E-02	Inhibited	-2.738	bias		CCRL2,CEBPD,CXCL1,FPR2,IL1RN,ITGAM,PTGS2,RNF149
E. coli serotype 0127B8 lipopolysaccharide		chemical - endogenous non-mammalian	1.28E-03	1.65E-02	Activated	2.236		1.706	CCRL2,FPR2,MYD88,PTGS2,TLR4
SP4		transcription regulator	1.28E-03	1.65E-02					IL1RN,OSM,PRKCD
F2		peptidase	1.31E-03	1.67E-02	Activated	2.36	bias		BCL3,CXCL1,LAMP2,OSM,PTGS2,SLC2A3
fish oils		chemical drug	1.36E-03	1.72E-02					ACSL1,PTGS2,TLR4
rosiglitazone		chemical drug	1.35E-03	1.72E-02		0.847		0.227	ACSL1,ALOX5,CCRL2,GOT1,IL1RN,ITGAM,PTGS2,TLR4
RRP1B		transcription regulator	1.38E-03	1.74E-02					PRKCD,RPL6,RPS14,RPS3
PP2A		complex	1.42E-03	1.76E-02					LYN,PTGS2
YAP/TAZ		group	1.42E-03	1.76E-02					GOT1,PTGS2
poly-L-lysine		chemical reagent	1.42E-03	1.76E-02					IL1RN,ITGAM
topotecan		chemical drug	1.44E-03	1.78E-02		0.555	bias	1.175	ACSL1,BCL3,BTG1,PTGS2,USP10
RNA polymerase II		complex	1.46E-03	1.79E-02					GOT1,IL1RN,PTGS2,RPS3,SLC2A3,SPI1
IRF1		transcription regulator	1.48E-03	1.80E-02		1.941	bias		FPR2,IL17RA,PTGS2,SELL,SPI1
SMAD4		transcription regulator	1.60E-03	1.94E-02					CAB39,ETV6,IRAK3,PILRA,PTGS2,SPI1
salicylic acid		chemical drug	1.61E-03	1.95E-02					FKBP5,PTGS2,TLR4
Collagen Alpha1		group	1.66E-03	1.96E-02					IL1RN,PTGS2
PLCE1		enzyme	1.66E-03	1.96E-02					CXCR2,PTGS2
ebselen		chemical drug	1.66E-03	1.96E-02					PTGS2,TLR4
loxoribine		chemical drug	1.66E-03	1.96E-02					PTGS2,TLR4
growth factor		group	1.66E-03	1.96E-02					PTGS2,SELL
platelet activating factor		chemical - endogenous mammalian	1.70E-03	1.99E-02					ITGAM,PTGS2,SELL
N-[N-(3,5-difluorophenacetyl-L-Ala)]-S-phenylglycine t-butyl ester		chemical - protease inhibitor	1.70E-03	1.99E-02					CXCL1,MYD88,TLR4
NUPR1		transcription regulator	1.72E-03	2.00E-02		0.707		1.337	BTG1,CREB5,ETV6,MEGF9,MYD88,PARP1,PFKFB3,TMEM167B
Tlr		group	1.73E-03	2.01E-02					CXCR2,FPR2,IRAK3,PTGS2
diphtheria toxin		chemical - endogenous non-mammalian	1.79E-03	2.04E-02					BCL3,GCA,LRRK2,PFKFB3
HDL		complex	1.79E-03	2.04E-02					ITGAM,MYD88,PTGS2
HIF1A		transcription regulator	1.78E-03	2.04E-02		1.705	bias	0.689	CCRL2,CYP4F3,FAM53C,IL17RA,NFKBIZ,PFKFB3,PTGS2,SLC2A3
carbonyl cyanide m-chlorophenyl hydrazone		chemical toxicant	1.79E-03	2.04E-02					NFKBIZ,PTGS2,TMEM154
PTPRJ		phosphatase	1.89E-03	2.09E-02					CEBPD,NFKBIZ,PTGS2
BDKRB2		G-protein coupled receptor	1.91E-03	2.09E-02					ITGAM,PTGS2
SMPD1		enzyme	1.89E-03	2.09E-02					CCRL2,CXCL1,MYD88
BTC		growth factor	1.91E-03	2.09E-02					ITGAM,PTGS2
Z-551		chemical reagent	1.91E-03	2.09E-02					ITGAM,TLR4
paraquat		chemical toxicant	1.90E-03	2.09E-02					CEBPD,ITGAM,LAMP2,PTGS2
dexmedetomidine		chemical drug	1.91E-03	2.09E-02					MYD88,PTGS2
chlorogenic acid		chemical drug	1.91E-03	2.09E-02					PTGS2,TLR4

bicuculline		chemical - endogenous non-mammalian	1.99E-03	2.17E-02					PFKFB3,PTGS2,SLC2A3
PI3K (complex)		complex	2.00E-03	2.18E-02	Activated	2.201	bias		IL1RN,ITGAM,PTGS2,SELL,SPI1,TLR4
IRF6		transcription regulator	2.09E-03	2.25E-02					FPR2,PTGS2,TLR4
CpG ODN 1668		chemical reagent	2.09E-03	2.25E-02					IRAK3,PTGS2,TLR4
3M-002		chemical reagent	2.14E-03	2.27E-02					FPR1,MYD88,PTGS2,TLR8
FCGR1A		transmembrane receptor	2.17E-03	2.27E-02					IL1RN,ITGAM
CXCL2		cytokine	2.17E-03	2.27E-02					CXCR2,ITGAM
101.10 peptide		chemical reagent	2.17E-03	2.27E-02					IL1RN,PTGS2
Go6983		chemical - kinase inhibitor	2.17E-03	2.27E-02					PRKCD,PTGS2
hydrogen sulfide		chemical - endogenous mammalian	2.17E-03	2.27E-02					PTGS2,SELL
caffeic acid		chemical drug	2.17E-03	2.27E-02					PTGS2,TLR4
VEGFA		growth factor	2.24E-03	2.34E-02		0.582	bias		ACSL1,CCRL2,PARP1,PTGS2,SHMT2,TLR1
STAT5a/b		group	2.27E-03	2.36E-02					MYD88,OSM,PTGS2,SLC2A3
CD44		other	2.29E-03	2.37E-02		1.964	bias		FKBP5,IL1RN,IRAK3,SELL,TLR8
dalfampridine		chemical drug	2.30E-03	2.37E-02					PFKFB3,PTGS2,SLC2A3
oleic acid		chemical - endogenous mammalian	2.34E-03	2.40E-02		-0.927		-1.199	ACSL1,ALOX5,PTGS2,TLR4
HMOX1		enzyme	2.40E-03	2.45E-02		-0.689		-0.451	CXCL1,IL1RN,ITGAM,PTGS2
CHD1		enzyme	2.46E-03	2.46E-02					CXCL1,PTGS2
PSMB8		peptidase	2.46E-03	2.46E-02					PTGS2,RPS3
LBP		transporter	2.46E-03	2.46E-02					CXCR2,IL1RN
3-aminobenzamide		chemical toxicant	2.46E-03	2.46E-02					CXCL1,PARP1
clenbuterol		chemical drug	2.46E-03	2.46E-02					CXCL1,IL1RN
SP600125		chemical - kinase inhibitor	2.59E-03	2.58E-02		-0.588		-0.04	ALOX5,CXCL1,LAPTM5,PARP1,PTGS2,TLR4
ETV6-RUNX1		fusion gene/product	2.68E-03	2.66E-02	Inhibited	-2.216	bias		ALOX5,BCL3,LYN,MME,SELL,TLR4
glycine		chemical - endogenous mammalian	2.75E-03	2.66E-02					ITGAM,TLR4
Fcgr3		group	2.75E-03	2.66E-02					IL1RN,ITGAM
antimycin A		chemical - endogenous non-mammalian	2.75E-03	2.66E-02					SELL,TLR4
superoxide		chemical - endogenous mammalian	2.75E-03	2.66E-02					PTGS2,TLR8
rotenone		chemical toxicant	2.77E-03	2.66E-02					ITGAM,PTGS2,SELL
Sb202190		chemical - kinase inhibitor	2.76E-03	2.66E-02		-0.948	bias		ALOX5,FPR2,PTGS2,SPI1
magnolol		chemical - endogenous non-mammalian	2.75E-03	2.66E-02					PTGS2,TLR4
glycyrrhetinic acid		chemical drug	2.75E-03	2.66E-02					MYD88,TLR4
cholecalciferol		chemical - endogenous mammalian	2.84E-03	2.72E-02		1.963	bias	1.134	ALOX5,CEBPD,FCAR,ITGAM
NKX2-3		transcription regulator	2.85E-03	2.72E-02		-1.342		-1.379	BTG1,GCA,MYD88,PTGS2,SHMT2
tributyrin		chemical drug	2.89E-03	2.74E-02					MME,PTGS2,SLC30A1
F2RL1		G-protein coupled receptor	2.89E-03	2.74E-02					CXCL1,FPR2,PTGS2
WNT5A		cytokine	2.91E-03	2.74E-02		0.79		0.63	CXCL1,PTGS2,TLR1,TLR4
Tcf7		transcription regulator	2.91E-03	2.74E-02		0.555		0.61	LYN,MYD88,PARP1,SELL,TLR1
PHLPP1		enzyme	3.07E-03	2.83E-02					CEBPD,PRKCD
n-nitrosomethylbenzylamine		chemical toxicant	3.07E-03	2.83E-02					LAMP2,NDEL1,PTGS2,SLC30A1
U0126		chemical - kinase inhibitor	3.04E-03	2.83E-02		-1.144	bias	0.083	CEBPD,CXCL1,CYP4F3,FKBP5,LAMP2,NUMB,PTGS2,SLC2A3
PS-1145		chemical - kinase inhibitor	3.07E-03	2.83E-02					CXCL1,PTGS2
helenalin		chemical - endogenous non-mammalian	3.07E-03	2.83E-02					CXCL1,PTGS2
peroxynitrite		chemical toxicant	3.07E-03	2.83E-02					LYN,PTGS2
ATP-gamma-S		chemical reagent	3.15E-03	2.89E-02					BTG1,PTGS2,TLR1
IL22		cytokine	3.24E-03	2.95E-02		1.972	bias	1.152	BCL3,CXCL1,CXCR2,PTGS2
15-deoxy-delta-12-14 prostaglandin D2		chemical - endogenous mammalian	4.36E-03	3.00E-02					ITGAM
17-phenyl-18,19,20-trinor-prostaglandin d2		chemical reagent	4.36E-03	3.00E-02					ITGAM
Synuclein		group	4.36E-03	3.00E-02					LRRK2
thromboxane A2		chemical - endogenous mammalian	4.36E-03	3.00E-02					PTGS2
kawain		chemical - endogenous non-mammalian	3.40E-03	3.00E-02					MYD88,TLR4
etalocib		chemical drug	4.36E-03	3.00E-02					ITGAM
hexacosanoyl-coenzyme A		chemical - endogenous non-mammalian	4.36E-03	3.00E-02					ALOX5
GÎ±q		group	4.36E-03	3.00E-02					PTGS2
beta-glucuronidase		group	4.36E-03	3.00E-02					PTGS2
PDGF BB		complex	3.46E-03	3.00E-02	Activated	2.203	bias	1.268	BCL3,CEBPD,PTGS2,RPS14,SLC2A3,SPI1
3',5'-methoxyflavone		chemical reagent	4.36E-03	3.00E-02					PTGS2
N-formyl-Nle-Leu-Phe		chemical reagent	4.36E-03	3.00E-02					FPR1
MAP2K4/7		group	4.36E-03	3.00E-02					PTGS2

DOT1L		phosphatase	4.11E-03	3.00E-02					IRAK3,SPI1
Pad2		other	4.36E-03	3.00E-02					PTGS2
IDH3B		enzyme	4.36E-03	3.00E-02					PFKFB3
RNF31		enzyme	4.17E-03	3.00E-02					CXCL1,IL18RAP,IL1RN
artesunic acid		chemical drug	3.40E-03	3.00E-02					PTGS2,TLR4
procyanidin B2		chemical - endogenous non-mammalian	4.36E-03	3.00E-02					PTGS2
LMAN1		other	4.36E-03	3.00E-02					F5
SIRT6		enzyme	3.71E-03	3.00E-02					IRAK3,PTGS2,RPL6
Growth hormone		group	4.34E-03	3.00E-02	Activated	2	bias	1.332	ACSL1,CEBPD,CXCL1,FKBP5,PFKFB3
nickel sulfide		chemical reagent	4.36E-03	3.00E-02					PTGS2
Tpl2 kinase inhibitor		chemical - kinase inhibitor	4.36E-03	3.00E-02					PTGS2
CL097		chemical reagent	3.40E-03	3.00E-02					BCL3,IRAK3
AZD1981		chemical drug	4.36E-03	3.00E-02					ITGAM
PARG		enzyme	3.75E-03	3.00E-02					PARP1,PTGS2
BCOR		transcription regulator	3.40E-03	3.00E-02					ETV6,TLR4
cucurbitacin E		chemical - endogenous non-mammalian	4.36E-03	3.00E-02					PTGS2
PTEN		phosphatase	3.93E-03	3.00E-02		-1.31		-1.249	ACSL1,BCL3,BTG1,LAMP2,MME,PARP1,PFKFB3,PTGS2,SELL
CYP2E1		enzyme	3.75E-03	3.00E-02					PTGS2,TLR4
EDN2		growth factor	4.36E-03	3.00E-02					PTGS2
IRF8		transcription regulator	3.67E-03	3.00E-02					ETV6,IL17RA,ITGAM,TLR4
ACOX1		enzyme	3.76E-03	3.00E-02		-0.577		-0.344	ACSL1,GOT1,HAL,QPCT
mir-223		microRNA	3.67E-03	3.00E-02		1.934		1.708	BST1,IL1RN,PARP1,TLR4
mir-181		microRNA	3.86E-03	3.00E-02					MME,PRKCD,PTGS2
mir-1906		microRNA	4.36E-03	3.00E-02					TLR4
miR-642a-5p (miRNAs w/seed UCCCUCU)		mature microRNA	4.36E-03	3.00E-02					SHMT2
SOS2		other	4.36E-03	3.00E-02					SELL
SIGLEC7		transmembrane receptor	4.36E-03	3.00E-02					PTGS2
SIGLEC9		other	4.36E-03	3.00E-02					PTGS2
SOCS6		other	3.75E-03	3.00E-02					CEBPD,PTGS2
CRH		cytokine	3.57E-03	3.00E-02					IL1RN,SLC2A3,TLR4
AZ 11645373		chemical reagent	4.36E-03	3.00E-02					SELL
5-methoxytryptophan		chemical - endogenous mammalian	4.36E-03	3.00E-02					PTGS2
TRAF3IP2		enzyme	4.02E-03	3.00E-02					CEBPD,NFKBIZ,STEAP4
APOE		transporter	3.41E-03	3.00E-02					ACSL1,FKBP5,IL1RN,ITGAM,PTGS2,SLC25A44
LUC7L3		other	4.36E-03	3.00E-02					PTGS2
GNA13		enzyme	3.75E-03	3.00E-02					CXCL1,PTGS2
TFF1		other	4.36E-03	3.00E-02					PTGS2
FASN		enzyme	3.43E-03	3.00E-02					ALOX5,LRRK2,PTGS2
Pla2g2a		other	4.36E-03	3.00E-02					PTGS2
THEM4		enzyme	4.36E-03	3.00E-02					PTGS2
IL15		cytokine	4.13E-03	3.00E-02		1.445	bias	0.646	BTG1,GNL2,IL18RAP,LYN,MYD88,PFKFB3,SELL
CISH		other	4.17E-03	3.00E-02					CCRL2,OSM,PTGS2
ZFPM1		transcription regulator	4.02E-03	3.00E-02					ALOX5,IRAK3,SPI1
PRC1		other	4.36E-03	3.00E-02					PTGS2
IKZF3		transcription regulator	3.71E-03	3.00E-02					ALOX5,NFKBIZ,TLR4
IL7		cytokine	4.05E-03	3.00E-02		0.577	bias	-0.245	CXCL1,IL18RAP,LYN,SELL
OSM	0.521	cytokine	3.31E-03	3.00E-02		1.948	bias	1.263	BCL3,CEBPD,CXCL1,CYP4F3,GCA,MYD88,OSM,PFKFB3
GABRA2		ion channel	4.36E-03	3.00E-02					TLR4
ZNF433		transcription regulator	4.36E-03	3.00E-02					ALOX5
MTARC2		enzyme	4.36E-03	3.00E-02					MTARC1
PTPN14		phosphatase	4.36E-03	3.00E-02					PRKCD
prexasertib		chemical drug	3.43E-03	3.00E-02					BCL3,OSM,PTGS2
PEX14		transcription regulator	4.36E-03	3.00E-02					PTGS2
CGP 52608		chemical reagent	4.36E-03	3.00E-02					ALOX5
S-methylisothiopseudouronium		chemical reagent	4.36E-03	3.00E-02					PTGS2
LY311727		chemical reagent	4.36E-03	3.00E-02					PTGS2
BN 50730		chemical reagent	4.36E-03	3.00E-02					PTGS2
1-(1-glycero)dodeca-1,3,5,7,9-pentaene		chemical toxicant	4.36E-03	3.00E-02					PTGS2
aceclofenac		chemical drug	4.36E-03	3.00E-02					SELL
methotrexate		chemical drug	4.34E-03	3.00E-02					CXCR2,FPR1,FPR2,PTGS2,SELL
D-lactic acid		chemical - endogenous mammalian	4.36E-03	3.00E-02					ITGAM
Ro 31-7549		chemical - kinase inhibitor	4.36E-03	3.00E-02					PTGS2
3,4-dihydro-5-[4-(1-piperidinyl)butoxy]-1(2H)-isoquinolinone		chemical reagent	4.36E-03	3.00E-02					PARP1
theaflavin monogallate B		chemical - endogenous non-mammalian	4.36E-03	3.00E-02					PTGS2
4-cresol		chemical toxicant	4.36E-03	3.00E-02					ITGAM
PAR-1-specific peptide		chemical reagent	4.36E-03	3.00E-02					OSM

N-t-Boc-Phe-D-Leu-Phe-D-Leu-Phe		chemical reagent	4.36E-03	3.00E-02					PTGS2
limonoate D-ring-lactone		chemical - endogenous non-mammalian	4.36E-03	3.00E-02					TLR4
triptolide		chemical drug	3.67E-03	3.00E-02					CXCL1,CXCR2,PTGS2,TLR4
anti-mir-642a-5p inhibitor		chemical reagent	4.36E-03	3.00E-02					SHMT2
tyrphostin AG 1478		chemical - kinase inhibitor	4.02E-03	3.00E-02					ITGAM,MME,PTGS2
n-6 docosapentaenoic acid		chemical - other	4.36E-03	3.00E-02					PTGS2
long-chain alcohol		chemical - endogenous non-mammalian	4.36E-03	3.00E-02					PTGS2
BX-912		chemical - kinase inhibitor	4.36E-03	3.00E-02					ITGAM
P2y Receptor		group	4.36E-03	3.00E-02					SLC2A3
2-deoxyglucose		chemical drug	4.02E-03	3.00E-02					GOT1,PTGS2,SPI1
metribolone		chemical reagent	3.36E-03	3.00E-02		1.299	bias		ATP6V0B,FKBP5,FPR1,GOT1,PDK3,PRKCD,TM9SF2
prednisolone		chemical drug	4.50E-03	3.04E-02		0.365	bias	1.074	BTG1,CEBPD,FKBP5,ITGAM,PTGS2
NADPH oxidase		complex	4.49E-03	3.04E-02					CXCL1,PTGS2
STK40		kinase	4.50E-03	3.04E-02					CXCL1,PTGS2,TLR4
SCGB1A1		cytokine	4.49E-03	3.04E-02					PTGS2,SELL
minocycline		chemical drug	4.50E-03	3.04E-02					ALOX5,ITGAM,PTGS2
epicatechin gallate		chemical drug	4.49E-03	3.04E-02					LYN,PTGS2
MAP3K8		kinase	4.66E-03	3.14E-02		0.954		0.745	CEBPD,GCA,NFAM1,PTGS2
cerivastatin		chemical drug	4.66E-03	3.14E-02					AGTRAP,ETV6,PTGS2
NR3C1		ligand-dependent nuclear receptor	4.75E-03	3.18E-02		1		0.799	BTG1,CEBPD,FKBP5,MYD88,PFKFB3,PRKCD,PTGS2,SLC2A3,TLR1
IL1A		cytokine	4.81E-03	3.22E-02	Activated	2.179	bias		BCL3,CXCL1,IL1RN,NFKBIZ,PTGS2
2-aminopurine		chemical reagent	4.88E-03	3.23E-02					CXCL1,PTGS2
IL36B		cytokine	4.88E-03	3.23E-02					CXCL1,NFKBIZ
RBP4		other	4.88E-03	3.23E-02					MYD88,TLR4
diosgenin		chemical - endogenous non-mammalian	4.88E-03	3.23E-02					ALOX5,PTGS2
CCL5		cytokine	5.01E-03	3.28E-02					CCRL2,ITGAM,TLR4
(+)-MK-801		chemical drug	5.01E-03	3.28E-02					ITGAM,PTGS2,SELL
rottlerin		chemical - kinase inhibitor	5.01E-03	3.28E-02					FKBP5,PRKCD,PTGS2
PLX5622		chemical drug	5.01E-03	3.28E-02					IL1RN,OSM,TLR4
ZBTB10		other	5.21E-03	3.41E-02	Activated	2	bias		IL1RN,LRRK2,MYD88,PTGS2
MTOR		kinase	5.24E-03	3.42E-02		-0.152		-0.581	CEBPD,CXCL1,PDK3,RPS18,SELL,TLR4
calcipotriene		chemical drug	5.29E-03	3.43E-02					PRKCD,PTGS2
COMMD1		transporter	5.29E-03	3.43E-02					PFKFB3,TLR1
MAP3K14		kinase	5.37E-03	3.47E-02					BST1,CXCL1,PTGS2
leukotriene B4		chemical - endogenous mammalian	5.72E-03	3.63E-02					ITGAM,MYD88
STAT1		transcription regulator	5.67E-03	3.63E-02					BTG1,CCRL2,CEBPD,PTGS2,TLR4,TLR8
MST1		growth factor	5.72E-03	3.63E-02					IL1RN,PTGS2
XIAP		enzyme	5.72E-03	3.63E-02					CXCL1,PTGS2
BAG1		other	5.72E-03	3.63E-02					FKBP5,PTGS2
WT1		transcription regulator	5.67E-03	3.63E-02					IL1RN,ITGAM,NCSTN,OSM,SLC2A3
L 663536		chemical reagent	5.72E-03	3.63E-02					ALOX5,NCSTN
Bay 11-7082		chemical - kinase inhibitor	5.75E-03	3.64E-02					CXCL1,FPR2,PTGS2
PP2/AG1879 tyrosine kinase inhibitor		chemical - kinase inhibitor	5.94E-03	3.75E-02					ITGAM,LYN,PTGS2
TNFRSF1A		transmembrane receptor	5.94E-03	3.75E-02		0.152		-0.194	CXCL1,ITGAM,SELL,TLR4
AGT		growth factor	6.08E-03	3.82E-02		1.57	bias	0.608	CR1,CREB5,FKBP5,LAMP2,PARP1,PTGS2,SELL,SPI1,SSH2,TLR1
CD300LF		other	6.16E-03	3.85E-02					IL1RN,SLC2A3
JINK1/2		group	6.16E-03	3.85E-02					PARP1,PTGS2
P38 MAPK		group	6.20E-03	3.87E-02	Activated	2.134	bias		CEBPD,CXCL1,CYP4F3,IL1RN,ITGAM,PTGS2
cyclic AMP		chemical - endogenous mammalian	6.63E-03	4.08E-02		1.334	bias	0.295	CEBPD,GOT1,IL1RN,MME,PTGS2
NOTCH1		transcription regulator	6.63E-03	4.08E-02		-0.577		-0.74	CEBPD,CXCL1,ITGAM,PTGS2,SPI1
CARD11		kinase	6.61E-03	4.08E-02					MME,SPI1
JAK inhibitor I		chemical - kinase inhibitor	6.61E-03	4.08E-02					CXCL1,PTGS2
phorbol 12,13-didecanoate		chemical toxicant	6.61E-03	4.08E-02					PARP1,PRKCD
TAC1		other	6.76E-03	4.15E-02					IL1RN,MME,PTGS2
curcumin		chemical drug	6.84E-03	4.20E-02		-1.758		-1.235	ALOX5,CR1,CXCL1,ITGAM,PTGS2,TLR4
FGF19		growth factor	6.97E-03	4.27E-02					ACSL1,IL1RN,MME
fluticasone propionate		chemical drug	7.00E-03	4.27E-02		0.577		0.531	ALOX5,CXCL1,FKBP5,IL1RN
ELF1		transcription regulator	7.08E-03	4.30E-02					FCAR,SPI1
hemozoin		chemical - endogenous non-mammalian	7.08E-03	4.30E-02					CXCL1,CXCR2
spironolactone		chemical drug	7.19E-03	4.36E-02					FKBP5,PTGS2,SELL
prostaglandin D2		chemical - endogenous mammalian	7.57E-03	4.39E-02					ITGAM,PTGS2
15-keto-13,14-dihydroprostaglandin E2		chemical - endogenous mammalian	8.71E-03	4.39E-02					OSM
arbutin		chemical drug	8.71E-03	4.39E-02					PTGS2

1alpha,25-dihydroxy-previtamin D3		chemical reagent	8.71E-03	4.39E-02					PRKCD
hydrogen peroxide		chemical - endogenous mammalian	8.31E-03	4.39E-02		1.708	bias	0.779	ALOX5,PARP1,PTGS2,RPL5,RTN3,SLC30A1,TLR4
prostaglandin E3		chemical - endogenous mammalian	8.71E-03	4.39E-02					PTGS2
HP1		group	8.71E-03	4.39E-02					PTGS2
Steroid 5 alpha-Reductase		group	8.71E-03	4.39E-02					PTGS2
anakinra		biologic drug	8.07E-03	4.39E-02					CXCL1,PTGS2
MEF2		group	8.07E-03	4.39E-02					IL1RN,PTGS2
NPS		other	8.71E-03	4.39E-02					ITGAM
Calcb		other	8.71E-03	4.39E-02					SPI1
sPla2		group	8.71E-03	4.39E-02					PTGS2
IFN Beta		group	7.73E-03	4.39E-02		0.771	bias	-0.013	CEBPD,CXCL1,IL1RN,TLR4
GSDMB		other	8.71E-03	4.39E-02					ALOX5
LPAR5		G-protein coupled receptor	8.71E-03	4.39E-02					PTGS2
VRK2		kinase	8.71E-03	4.39E-02					PTGS2
ZC3H12C		other	8.58E-03	4.39E-02					FPR2,TLR8
CLEC7A		transmembrane receptor	7.57E-03	4.39E-02					PTGS2,TLR4
veliparib		chemical drug	8.71E-03	4.39E-02					PARP1
3,4-dihydroxyphenylethanol		chemical drug	8.71E-03	4.39E-02					PTGS2
empagliflozin		chemical drug	8.71E-03	4.39E-02					TLR4
IKBKE		kinase	7.64E-03	4.39E-02					PDK3,PTGS2,SLC2A3
ZFP36		transcription regulator	7.64E-03	4.39E-02					OSM,PTGS2,TLR4
RPSA		translation regulator	7.57E-03	4.39E-02					PTGS2,TLR4
PLA2G2F		enzyme	8.71E-03	4.39E-02					PTGS2
FOXP3		transcription regulator	8.34E-03	4.39E-02					IL17RA,IL18RAP,SELL,SLC2A3
miR-489-3p (miRNAs w/seed UGACAUC)		mature microRNA	8.71E-03	4.39E-02					GCA
MIR585		microRNA	8.71E-03	4.39E-02					PARP1
2-(4-amino-1-isopropyl-1H-pyrazolo[3,4-d]pyrimidin-3-yl)-1H-indol-5-ol		chemical reagent	8.34E-03	4.39E-02		1.98		2.134	BTG1,IL1RN,PARP1,PTGS2
potassium nitrate		chemical drug	8.71E-03	4.39E-02					ITGAM
IRF7		transcription regulator	7.29E-03	4.39E-02	Activated	2	bias		CCRL2,ITGAM,TLR4,TLR8
GATAD2B		transcription regulator	8.71E-03	4.39E-02					PTGS2
Tpi(6)-Leu(13)-psi(CH2NH)-Leu(14)-bombesin (6-14)		chemical reagent	8.71E-03	4.39E-02					ITGAM
FAAH		enzyme	7.57E-03	4.39E-02					RPL5,RPS18
LTB4R		G-protein coupled receptor	7.57E-03	4.39E-02					ALOX5,PTGS2
APEX1		enzyme	8.58E-03	4.39E-02					ALOX5,PTGS2
UXT		transcription regulator	8.58E-03	4.39E-02					F5,FKBP5
CFP		other	8.71E-03	4.39E-02					ITGAM
CBR1		enzyme	8.71E-03	4.39E-02					PTGS2
VE-821		chemical reagent	8.71E-03	4.39E-02					ITGAM
TCF7L2		transcription regulator	8.57E-03	4.39E-02	Activated	2.449	bias		ACSL1,ALOX5,CREB5,KLHL2,MOSPD2,PTGS2
ARHGDIB		enzyme	8.71E-03	4.39E-02					PTGS2
HDAC1		transcription regulator	8.39E-03	4.39E-02					AGTRAP,CXCL1,MME,PFKFB3,PTGS2
VTN		other	8.07E-03	4.39E-02					ITGAM,PTGS2
RECQL5		enzyme	8.71E-03	4.39E-02					PARP1
EHF		transcription regulator	8.58E-03	4.39E-02					ALOX5,IL1RN,LYN
brequinar		chemical drug	8.71E-03	4.39E-02					ITGAM
Hbb-b1		transporter	7.87E-03	4.39E-02					CXCR2,ITGAM,SPI1
SH3GLB2		other	8.71E-03	4.39E-02					PTGS2
doxycycline		chemical drug	7.87E-03	4.39E-02					CEBPD,PTGS2,SPI1
semapimod		chemical drug	8.71E-03	4.39E-02					PTGS2
SC68376		chemical - kinase inhibitor	8.71E-03	4.39E-02					PTGS2
gefitinib		chemical drug	7.73E-03	4.39E-02					CEBPD,OSM,PTGS2,SLC2A3
neocuproine		chemical reagent	8.71E-03	4.39E-02					CXCL1
pyridoxal		chemical - endogenous mammalian	8.71E-03	4.39E-02					PTGS2
bifenthrin		chemical toxicant	8.71E-03	4.39E-02					PTGS2
mefenamic acid		chemical drug	8.71E-03	4.39E-02					SELL
benzylamine		chemical - endogenous mammalian	8.71E-03	4.39E-02					PTGS2
aspirin		chemical drug	7.58E-03	4.39E-02		0.727	bias	1.584	CXCL1,LAMP2,PTGS2,SELL
CGP77675		chemical - kinase inhibitor	8.71E-03	4.39E-02					PTGS2
bumetanide		chemical drug	8.71E-03	4.39E-02					PTGS2
gliclazide		chemical drug	8.71E-03	4.39E-02					SLC2A3
ramipril		chemical drug	7.57E-03	4.39E-02					PTGS2,TLR4
biliverdine		chemical - endogenous mammalian	8.71E-03	4.39E-02					TLR4
methylprednisolone acetate		chemical drug	8.71E-03	4.39E-02					PTGS2
vanillic acid		chemical - endogenous mammalian	8.71E-03	4.39E-02					PTGS2

nexinhib4		chemical reagent	8.71E-03	4.39E-02					ITGAM
(-)-epigallocatechin-3-O-(3''-O-methyl)-gallate		chemical - endogenous non-mammalian	8.71E-03	4.39E-02					TLR4
AJH-836		chemical reagent	8.71E-03	4.39E-02					PRKCD
2-hydroxyarachidonic acid		chemical reagent	8.71E-03	4.39E-02					PTGS2
Alocasia cucullata root extract		chemical reagent	8.71E-03	4.39E-02					ITGAM
trinitrobenzenesulfonic acid		chemical reagent	8.10E-03	4.39E-02					BST1,ITGAM,PTGS2
desmopressin		biologic drug	8.10E-03	4.39E-02					MME,PLXDC2,RPS3
ceruletide		biologic drug	8.07E-03	4.39E-02					MME,PTGS2
nucleoside		chemical - endogenous mammalian	8.71E-03	4.39E-02					ITGAM
phorbol esters		chemical - other	7.41E-03	4.39E-02					ITGAM,OSM,PTGS2
betulinic acid		chemical drug	8.58E-03	4.39E-02					PTGS2,TLR4
tetrahydropalmatine		chemical - endogenous non-mammalian	8.71E-03	4.39E-02					PTGS2
lipooligosaccharide		chemical toxicant	8.71E-03	4.39E-02					PTGS2
tibolone		chemical drug	8.71E-03	4.39E-02					PFKFB3
tripterine		chemical - endogenous non-mammalian	8.07E-03	4.39E-02					MYD88,TLR4
TCF		group	7.64E-03	4.39E-02					MME,PTGS2,QPCT
Ifn gamma		complex	8.83E-03	4.44E-02					IL17RA,MYD88,TLR4
POU2F2		transcription regulator	8.83E-03	4.44E-02					ALOX5,PFKFB3,SPI1
IL5		cytokine	8.87E-03	4.44E-02	Activated	2.148	bias		BCL3,CCRL2,CXCR2,ITGAM,TLR1
genistein		chemical drug	8.87E-03	4.44E-02		0.181		-0.253	ALOX5,ATP6V0B,BCL3,CXCR2,LAPTM5,PTGS2
Fgf		group	9.11E-03	4.48E-02					GNL3,PTGS2
3M-011		chemical reagent	9.11E-03	4.48E-02					MYD88,PTGS2
IKBKB		kinase	9.00E-03	4.48E-02		1.965	bias	1.09	CEBPD,CXCL1,IL1RN,MYD88,PTGS2
miR-125b-5p (and other miRNAs w/seed CCCUGAG)		mature microRNA	9.08E-03	4.48E-02					ALOX5,IL1RN,MYD88
YBX1		transcription regulator	9.08E-03	4.48E-02					OSM,PTGS2,SLC2A3
APP		other	8.98E-03	4.48E-02	Activated	2.323	bias	1.618	CXCL1,CXCR2,ITGAM,MME,MYD88,OSM,PFKFB3,PTGS2,TLR4,TLR8
WNT4		cytokine	9.11E-03	4.48E-02					FKBP5,PTGS2
MARK2		kinase	9.11E-03	4.48E-02					NFKBIZ,PTGS2
ATG7		enzyme	9.08E-03	4.48E-02					ACSL1,PRKCD,TLR4
NCOR1		transcription regulator	9.33E-03	4.57E-02					BCL3,MYD88,PTGS2
IRF4		transcription regulator	9.32E-03	4.57E-02					IL17RA,IL18RAP,IL1RN,RPL6
NFKB1		transcription regulator	9.51E-03	4.65E-02					BCL3,IL1RN,PRKCD,PTGS2,SPI1
imiquimod		chemical drug	9.59E-03	4.68E-02					IL1RN,PTGS2,SELL
CHRNA7		transmembrane receptor	9.65E-03	4.69E-02					IL1RN,ITGAM
SELP		transmembrane receptor	9.65E-03	4.69E-02					BST1,PRKCD
eicosapentenoic acid		chemical drug	9.86E-03	4.78E-02					CEBPD,PTGS2,SPI1
TNFSF10		cytokine	1.01E-02	4.90E-02					IL1RN,ITGAM,PTGS2
IL36A		cytokine	1.02E-02	4.93E-02					CXCL1,NFKBIZ
NR1H2		ligand-dependent nuclear receptor	1.04E-02	5.01E-02					IL1RN,PTGS2,RPS14
AKT inhibitor VIII		chemical reagent	1.08E-02	5.16E-02					PARP1,SELL
CDKN2A		transcription regulator	1.07E-02	5.16E-02		1.067		1.208	CEBPD,CXCR2,GNL3,PTGS2,TLR4
NCSTN	0.189	peptidase	1.08E-02	5.16E-02					CEBPD,SPI1
phorbol 12,13-dibutyrate		chemical - endogenous non-mammalian	1.08E-02	5.16E-02					PRKCD,PTGS2
RORA		ligand-dependent nuclear receptor	1.09E-02	5.22E-02					CXCR2,GOT1,PTGS2,SLC2A3
lipoxin A4		chemical - endogenous mammalian	1.14E-02	5.35E-02					FPR2,PTGS2
3M-001		chemical drug	1.14E-02	5.35E-02					MYD88,PTGS2
TANK		other	1.14E-02	5.35E-02					NFKBIZ,PTGS2
ID3		transcription regulator	1.13E-02	5.35E-02					BCL3,CXCL1,GNL3,SELL
PIK3CG		kinase	1.12E-02	5.35E-02					IL17RA,TLR1,TLR8
PDPK1		kinase	1.14E-02	5.35E-02					ALOX5,SELL
exenatide		biologic drug	1.14E-02	5.35E-02					ITGAM,PTGS2
acyline		biologic drug	1.15E-02	5.42E-02					RPL5,RPL6,RPS18
compound SI-1		chemical reagent	1.30E-02	5.46E-02					FKBP5
compound SI-2		chemical reagent	1.30E-02	5.46E-02					FKBP5
sulprostone		chemical drug	1.30E-02	5.46E-02					PTGS2
Rp-8-Br-cGMPS		chemical - kinase inhibitor	1.30E-02	5.46E-02					ITGAM
ergosterol-5,8-peroxide		chemical - endogenous mammalian	1.30E-02	5.46E-02					MYD88
chloride		chemical - endogenous mammalian	1.30E-02	5.46E-02					PTGS2
Beta Secretase		group	1.30E-02	5.46E-02					MME
Mapk		group	1.18E-02	5.46E-02					CXCL1,IRAK3,PTGS2

cardamomin		chemical - endogenous non-mammalian	1.30E-02	5.46E-02					PTGS2
SAA		group	1.26E-02	5.46E-02					IL1RN,PTGS2
des-Arg(10)-kallidin		chemical reagent	1.30E-02	5.46E-02					PTGS2
dietary fat derivative		chemical drug	1.30E-02	5.46E-02					ITGAM
imperatorin		chemical - endogenous non-mammalian	1.30E-02	5.46E-02					PTGS2
MAGEA11		other	1.30E-02	5.46E-02					FKBP5
IL17RC		transmembrane receptor	1.30E-02	5.46E-02					STEAP4
TRIM41		other	1.30E-02	5.46E-02					PRKCD
PSMD14		peptidase	1.30E-02	5.46E-02					BCL3
ASH1L		transcription regulator	1.30E-02	5.46E-02					IRAK3
HERC2		enzyme	1.30E-02	5.46E-02					FBXL5
KLHL21		other	1.30E-02	5.46E-02					NFKBIZ
bis(4-hydroxycinnamoyl)methane		chemical - endogenous non-mammalian	1.30E-02	5.46E-02					TLR4
Eotaxin		group	1.30E-02	5.46E-02					ITGAM
pycnogenols		chemical drug	1.30E-02	5.46E-02					PTGS2
pinoresinol		chemical - endogenous non-mammalian	1.30E-02	5.46E-02					PTGS2
vistusertib		chemical drug	1.30E-02	5.46E-02					MYD88
GCM2		transcription regulator	1.30E-02	5.46E-02					OSM
COL3A1		other	1.30E-02	5.46E-02					IL1RN
TMOD1		enzyme	1.30E-02	5.46E-02					GNL3
PSMC5		transcription regulator	1.30E-02	5.46E-02					LYN
17alpha-hydroxyprogesterone caproate		chemical drug	1.30E-02	5.46E-02					PTGS2
miR-515-3p (and other miRNAs w/seed AGUGCCU)		mature microRNA	1.30E-02	5.46E-02					BTG1
PTGER2		G-protein coupled receptor	1.30E-02	5.46E-02					CXCR2,FPR1,PTGS2
FOXO4		transcription regulator	1.24E-02	5.46E-02					ITGAM,PRKCD,SELL
GPR34		G-protein coupled receptor	1.30E-02	5.46E-02					ITGAM
ARRB2		other	1.26E-02	5.46E-02					NFKBIZ,PTGS2
JAG2		growth factor	1.26E-02	5.46E-02					CXCL1,IL1RN
PAGR1		other	1.30E-02	5.46E-02					CEBPD
quercetin-3-O-glucuronide		chemical - endogenous non-mammalian	1.30E-02	5.46E-02					PTGS2
ERC1		other	1.30E-02	5.46E-02					PTGS2
EDN1		cytokine	1.19E-02	5.46E-02		1.02	bias		ITGAM,PRKCD,PTGS2,SELL
EHMT1		transcription regulator	1.24E-02	5.46E-02					ACSL1,CEBPD,MEGF9
AZD-1208		chemical drug	1.30E-02	5.46E-02					SELL
ketoprofen		chemical drug	1.30E-02	5.46E-02					SELL
temocapril		chemical reagent	1.30E-02	5.46E-02					PTGS2
AA-861		chemical reagent	1.30E-02	5.46E-02					PTGS2
pioglitazone		chemical drug	1.17E-02	5.46E-02		-1.141		-1.204	CEBPD,IL1RN,ITGAM,PTGS2
amifostine		chemical drug	1.30E-02	5.46E-02					PTGS2
rocuronium		chemical drug	1.30E-02	5.46E-02					PTGS2
ropivacaine		chemical drug	1.30E-02	5.46E-02					ITGAM
benzamide		chemical reagent	1.30E-02	5.46E-02					PARP1
incyclinide		chemical drug	1.30E-02	5.46E-02					PTGS2
pyrrolidine dithiocarbamate		chemical reagent	1.27E-02	5.46E-02					CXCL1,PTGS2,TLR4
nomifensine		chemical drug	1.30E-02	5.46E-02					OSM
betamethasone valerate		chemical drug	1.30E-02	5.46E-02					PTGS2
senexin B		chemical drug	1.30E-02	5.46E-02					CXCL1
nexinhib20		chemical reagent	1.30E-02	5.46E-02					ITGAM
potassium tetraperoxochromate		chemical reagent	1.30E-02	5.46E-02					TLR4
tenidap		chemical drug	1.30E-02	5.46E-02					PTGS2
acacetin		chemical - endogenous non-mammalian	1.30E-02	5.46E-02					PTGS2
physostigmine		chemical drug	1.30E-02	5.46E-02					ITGAM
poly(ADP-ribose)		chemical - endogenous mammalian	1.30E-02	5.46E-02					PTGS2
poly(C)RNA		chemical reagent	1.30E-02	5.46E-02					PTGS2
taurine		chemical - endogenous mammalian	1.26E-02	5.46E-02					GOT1,PTGS2
epiallopregnanolone		chemical - endogenous mammalian	1.30E-02	5.46E-02					PTGS2
L-alpha-hydroxyglutarate		chemical - endogenous mammalian	1.30E-02	5.46E-02					SELL
NfkB1-RelA		complex	1.32E-02	5.49E-02					CXCL1,PTGS2
EP300		transcription regulator	1.32E-02	5.49E-02		0.878	bias		IL17RA,IL18RAP,MME,PARP1,PRKCD,PTGS2
FCER1G		transmembrane receptor	1.32E-02	5.49E-02					IL1RN,PTGS2
fluvastatin		chemical drug	1.32E-02	5.49E-02					AGTRAP,PTGS2

anandamide		chemical - endogenous mammalian	1.32E-02	5.49E-02					NCSTN,PTGS2
black raspberry extract		chemical drug	1.33E-02	5.53E-02					LAMP2,NDEL1,SLC30A1
GNA15		enzyme	1.33E-02	5.53E-02					CEBPD,IL1RN,PTGS2
GRIN3A		ion channel	1.37E-02	5.63E-02					LRRK2,NSUN7,PTGS2
mir-10		microRNA	1.37E-02	5.63E-02					ALOX5,FKBP5,MYD88
tamoxifen		chemical drug	1.37E-02	5.63E-02		-0.832		-0.899	CEBPD,IL1RN,PTGS2,SLC2A3,TLR4
PP1		chemical - kinase inhibitor	1.38E-02	5.64E-02					IL1RN,PTGS2
conjugated linoleic acid		chemical drug	1.38E-02	5.64E-02					IL1RN,PTGS2
RNASEH2A		enzyme	1.38E-02	5.64E-02					PTGS2,TLR4
AHR		ligand-dependent nuclear receptor	1.37E-02	5.64E-02	Activated	2.177		1.692	ALOX5,GOT1,IL18RAP,ITGAM,PTGS2,STEAP4
4-methylnitrosoamino-1-(3-pyridinyl)-1-butanone		chemical toxicant	1.38E-02	5.64E-02					ALOX5,PTGS2
laminaran		chemical drug	1.38E-02	5.64E-02					CXCL1,PTGS2
D-glucose		chemical - endogenous mammalian	1.38E-02	5.64E-02		1.19		0.586	ACSL1,GOT1,PARP1,PRKCD,PTGS2,RPS14,RTN3,TLR4
KIT		transmembrane receptor	1.40E-02	5.69E-02					IL17RA,ITGAM,OSM
cyclosporin A		biologic drug	1.42E-02	5.75E-02	Inhibited	-2.15		-1.678	CXCL1,F5,FKBP5,LYN,PTGS2,SELL
CHUK		kinase	1.42E-02	5.76E-02		0.883	bias		CEBPD,IL1RN,NUMB,PTGS2
IL17F		cytokine	1.45E-02	5.83E-02					CXCL1,PTGS2
ibuprofen		chemical drug	1.45E-02	5.83E-02					ITGAM,PTGS2
SAHM1		chemical reagent	1.45E-02	5.83E-02					OSM,PFKFB3
S-nitrosoglutathione		chemical toxicant	1.45E-02	5.83E-02					ITGAM,PTGS2
GH1		growth factor	1.46E-02	5.89E-02					CEBPD,RPL6,SHMT2
SP3		transcription regulator	1.49E-02	5.98E-02					ACSL1,PRKCD,PTGS2,SLC2A3
USP22		peptidase	1.51E-02	6.05E-02					ITGAM,SPI1
ARHGAP21		other	1.51E-02	6.05E-02					NFKBIZ,PTGS2
Cdc42		enzyme	1.51E-02	6.05E-02					LAMP2,SPI1
Ige		complex	1.56E-02	6.21E-02	Activated	2	bias		ALOX5,BCL3,OSM,PILRA,PTGS2
TLR7		transmembrane receptor	1.56E-02	6.21E-02		1.98	bias		CREB5,CXCL1,MYD88,TMEM154
lipid A		chemical toxicant	1.65E-02	6.24E-02					LYN,PRKCD
estriol		chemical - endogenous mammalian	1.65E-02	6.24E-02					CXCR2,IL1RN
platelet activating factor-C16		chemical - endogenous mammalian	1.73E-02	6.24E-02					ITGAM
8-chloro-cAMP		chemical drug	1.73E-02	6.24E-02					PTGS2
sargramostim		biologic drug	1.73E-02	6.24E-02					ITGAM
repertaxin		chemical drug	1.73E-02	6.24E-02					ITGAM
Creb		group	1.65E-02	6.24E-02					ALOX5,CEBPD,ITGAM,PTGS2,SLC25A44
NU-7026		chemical - kinase inhibitor	1.73E-02	6.24E-02					FKBP5
Metalloprotease		group	1.73E-02	6.24E-02					ITGAM
stevioside		chemical - endogenous non-mammalian	1.73E-02	6.24E-02					PTGS2
AMPK		complex	1.60E-02	6.24E-02					IL1RN,PFKFB3,PTGS2
tylophorine		chemical drug	1.73E-02	6.24E-02					PTGS2
PHA-665752		chemical - kinase inhibitor	1.73E-02	6.24E-02					PTGS2
PSMB11		peptidase	1.67E-02	6.24E-02					CREB5,ITGAM,PTGS2
TR-RXR		complex	1.73E-02	6.24E-02					BCL3
nebivolol		chemical drug	1.73E-02	6.24E-02					PTGS2
ZXDC		transcription regulator	1.73E-02	6.24E-02					ITGAM
TSPAN33		other	1.73E-02	6.24E-02					PTGS2
Carlr		other	1.73E-02	6.24E-02					PTGS2
IKBIP		other	1.73E-02	6.24E-02					NFKBIZ
CUEDC2		other	1.73E-02	6.24E-02					SLC2A3
GATAD2A		transcription regulator	1.73E-02	6.24E-02					PTGS2
Eif2		complex	1.73E-02	6.24E-02					PTGS2
NXF1		other	1.73E-02	6.24E-02					SELL
ZNF467		transcription regulator	1.73E-02	6.24E-02					OSM
interferon beta-1a		biologic drug	1.71E-02	6.24E-02					FPR1,IL1RN,TLR1
CHFR		enzyme	1.73E-02	6.24E-02					PARP1
ZNF281		transcription regulator	1.58E-02	6.24E-02					OSM,PTGS2
STAT4		transcription regulator	1.70E-02	6.24E-02		1.982	bias		BCL3,IL18RAP,SLC2A3,TMEM167B
MUC2		other	1.73E-02	6.24E-02					PTGS2
mir-19		microRNA	1.72E-02	6.24E-02					ALOX5,CXCL1
miR-718 (miRNAs w/seed UUCCGCC)		mature microRNA	1.73E-02	6.24E-02					TLR4
STAT6		transcription regulator	1.61E-02	6.24E-02		0.943		0.739	ACSL1,BCL3,FKBP5,IL1RN,IRAK3,PTGS2
K-604		chemical drug	1.73E-02	6.24E-02					PTGS2
NFE2L2		transcription regulator	1.65E-02	6.24E-02		0.958	bias	0.016	ETV6,FKBP5,GOT1,IL1RN,PTGS2,SHMT2
anatabine		chemical - endogenous non-mammalian	1.73E-02	6.24E-02					PTGS2
RNASEH1		enzyme	1.73E-02	6.24E-02					RPS3

SOS1		other	1.73E-02	6.24E-02					SELL
RUVBL1		transcription regulator	1.72E-02	6.24E-02					CYP4F3,FAM53C
FER		kinase	1.73E-02	6.24E-02					PRKCD
RGD1560225		other	1.73E-02	6.24E-02					PTGS2
TAL1		transcription regulator	1.73E-02	6.24E-02					NFKBIZ,SLC2A3,SPI1,SSH2
NDRG1		kinase	1.65E-02	6.24E-02					CXCL1,PARP1
SKLB023		chemical reagent	1.73E-02	6.24E-02					PTGS2
AKAP13		other	1.73E-02	6.24E-02					FKBP5
CD40		transmembrane receptor	1.68E-02	6.24E-02					IL1RN,ITGAM,PTGS2,SELL
VPS26A		transporter	1.73E-02	6.24E-02					SNX27
NFIB		transcription regulator	1.72E-02	6.24E-02					CREB5,STEAP4
Mt3		other	1.73E-02	6.24E-02					CEBPD
Ewsr1		other	1.73E-02	6.24E-02					PARP1
CYP4V2		enzyme	1.73E-02	6.24E-02					LAMP2
PELP1		other	1.71E-02	6.24E-02					CXCL1,GCA,PTGS2
CAY10397		chemical reagent	1.73E-02	6.24E-02					PTGS2
(+)-epicatechin		chemical drug	1.73E-02	6.24E-02					PTGS2
1-(2-hydroxy-5-methylphenyl)-3-phenyl-1,3-propanedione		chemical reagent	1.73E-02	6.24E-02					CEBPD
1-butanol		chemical - endogenous non-mammalian	1.73E-02	6.24E-02					PTGS2
PTIO		chemical reagent	1.73E-02	6.24E-02					ITGAM
Ro31-8425		chemical - kinase inhibitor	1.73E-02	6.24E-02					PRKCD
TAN 67		chemical reagent	1.73E-02	6.24E-02					ITGAM
ethyl linoleate		chemical - endogenous mammalian	1.73E-02	6.24E-02					PTGS2
cadmium sulfate		chemical toxicant	1.73E-02	6.24E-02					PTGS2
vanadium pentoxide		chemical toxicant	1.73E-02	6.24E-02					PTGS2
1-eicosapentaenoylglycerol		chemical reagent	1.73E-02	6.24E-02					PTGS2
beta-carotene		chemical - endogenous mammalian	1.65E-02	6.24E-02					MTARC1,PTGS2
flavone		chemical - endogenous non-mammalian	1.73E-02	6.24E-02					PTGS2
staurosporine		chemical - kinase inhibitor	1.67E-02	6.24E-02					MME,PTGS2,SELL
erucic acid		chemical - endogenous non-mammalian	1.73E-02	6.24E-02					ALOX5
PRKCA		kinase	1.74E-02	6.27E-02					LAMP2,PRKCD,PTGS2
ETS1		transcription regulator	1.75E-02	6.29E-02					ITGAM,PARP1,SELL,SPI1
FGF1		growth factor	1.78E-02	6.36E-02					CXCL1,ITGAM,PTGS2
LIPE		enzyme	1.78E-02	6.36E-02					ACSL1,FKBP5,PTGS2
SRC		kinase	1.78E-02	6.36E-02					LAMP2,PRKCD,PTGS2
celecoxib		chemical drug	1.78E-02	6.36E-02					ALOX5,PTGS2,TLR4
SERPINA1		other	1.79E-02	6.37E-02					IL1RN,TLR4
TCF7		transcription regulator	1.79E-02	6.37E-02					CEBPD,SPI1
4-hydroxynonenal		chemical toxicant	1.79E-02	6.37E-02					MME,PTGS2
FOXO3		transcription regulator	1.81E-02	6.41E-02					BTG1,ITGAM,PRKCD,RTN3,SELL
let-7		microRNA	1.83E-02	6.49E-02		-1.931	bias		CEBPD,MYD88,PTGS2,TLR4
Lh		complex	1.87E-02	6.54E-02		-0.046	bias	-0.92	FKBP5,PTGS2,RPL5,RPL6,RPS18
PROC		peptidase	1.87E-02	6.54E-02					F5,TLR4
MIF		cytokine	1.86E-02	6.54E-02					IL17RA,PTGS2,TLR4
CDKN1B		kinase	1.86E-02	6.54E-02					AGTRAP,ITGAM,NDEL1
OLR1		transmembrane receptor	1.87E-02	6.54E-02					CXCR2,ITGAM
herbimycin		chemical - kinase inhibitor	1.87E-02	6.54E-02					CXCR2,PTGS2
NUP98-DDX10		fusion gene/product	1.87E-02	6.54E-02					ALOX5,PTGS2
n-3 fatty acids		chemical drug	1.87E-02	6.54E-02					ALOX5,PTGS2
valproic acid		chemical drug	1.88E-02	6.59E-02		-1.067		-1.139	ETV6,LAGE3,MME,PRKCD,PTGS2,SPI1
MECP2		transcription regulator	1.89E-02	6.62E-02					FKBP5,PTGS2,SLC2A3
SMARCA5		transcription regulator	1.93E-02	6.74E-02					BST1,FKBP5,LYN
dimethyl sulfoxide		chemical drug	1.93E-02	6.74E-02					ALOX5,ITGAM,SPI1
SCARB1		transporter	1.94E-02	6.75E-02					IL1RN,PTGS2
NSC 172285		chemical reagent	2.16E-02	6.81E-02					MYD88
15-deoxy-delta-12,14 -PGJ 2		chemical - endogenous mammalian	2.10E-02	6.81E-02		-0.386		-0.221	CEBPD,ITGAM,PARP1,PTGS2
beta-glucan		chemical drug	2.16E-02	6.81E-02					IL1RN
beta-estradiol		chemical - endogenous mammalian	2.00E-02	6.81E-02		1.952	bias	0.729	ACSL1,BTG1,CEBPD,CXCL1,F5,GNL3,IGSF6,LAGE3,LAMP2,LAPTM5,MME,MOSPD2,PARP1,PRKCD,PTGS2,PTTG1IP,SLC2A3,TLR4
Nfatc		group	2.16E-02	6.81E-02					PTGS2
globotriaosylceramide		chemical - endogenous mammalian	2.16E-02	6.81E-02					LAMP2
dibutyryl cGMP		chemical reagent	2.16E-02	6.81E-02					PTGS2
fludrocortisone		chemical drug	2.16E-02	6.81E-02					SELL

dienogest		chemical drug	2.16E-02	6.81E-02					PTGS2
saxagliptin		chemical drug	2.16E-02	6.81E-02					TLR4
NF-kappaB decoy		biologic drug	2.16E-02	6.81E-02					PTGS2
Cpla2		group	2.16E-02	6.81E-02					PTGS2
W146		chemical reagent	2.16E-02	6.81E-02					PTGS2
aurapten		chemical - endogenous non-mammalian	2.16E-02	6.81E-02					PTGS2
beta-sitosterol		chemical reagent	2.16E-02	6.81E-02					PTGS2
MARCHF3		other	2.16E-02	6.81E-02					CXCL1
TRIM58		enzyme	2.16E-02	6.81E-02					MYD88
BUD23		enzyme	2.16E-02	6.81E-02					FKBP5
ENTPD5		enzyme	2.16E-02	6.81E-02					PRKCD
NLRX1		other	2.01E-02	6.81E-02					MYD88,TLR1
FLVCR1		transporter	2.16E-02	6.81E-02					RPS14
betulin		chemical - endogenous non-mammalian	2.16E-02	6.81E-02					PTGS2
RSF1		transcription regulator	2.16E-02	6.81E-02					PTGS2
GPR68		G-protein coupled receptor	2.16E-02	6.81E-02					PTGS2
PU-H71		chemical drug	2.16E-02	6.81E-02					LYN
carvacrol		chemical - endogenous non-mammalian	2.16E-02	6.81E-02					PTGS2
panobinostat		chemical drug	2.17E-02	6.81E-02					CEBPD,PTGS2
Mucin		group	2.16E-02	6.81E-02					PTGS2
EIF3I		translation regulator	2.16E-02	6.81E-02					PTGS2
Atf		group	2.16E-02	6.81E-02					PTGS2
DDX21		enzyme	2.16E-02	6.81E-02					RPS3
CCRL2	0.24	G-protein coupled receptor	2.16E-02	6.81E-02					CXCR2
IRF2		transcription regulator	2.05E-02	6.81E-02					PFKFB3,PTGS2,TLR4
LOC102724788/PRODH		enzyme	2.16E-02	6.81E-02					PTGS2
miR-292b-5p (and other miRNAs w/seed CUCAAAA)		mature microRNA	2.16E-02	6.81E-02					BTG1
NOS3		enzyme	2.01E-02	6.81E-02					PTGS2,TLR4
LDLR		transporter	1.99E-02	6.81E-02					FPR1,FPR2,IL1RN,TLR1
FUT4		enzyme	2.16E-02	6.81E-02					PARP1
ARLNC1		other	2.16E-02	6.81E-02					FKBP5
CDH4		other	2.16E-02	6.81E-02					PTGS2
LAMP1		other	2.16E-02	6.81E-02					LAMP2
NME2		kinase	2.16E-02	6.81E-02					PTGS2
RTN4R		transmembrane receptor	2.16E-02	6.81E-02					PTGS2
KAT2B		transcription regulator	2.01E-02	6.81E-02					ITGAM,PTGS2
RPTOR		other	2.01E-02	6.81E-02					BCL3,ITGAM,SPI1
URI1		transcription regulator	2.16E-02	6.81E-02					FKBP5
LRP1		transmembrane receptor	2.01E-02	6.81E-02					PTGS2,SLC2A3
DNM1		enzyme	2.16E-02	6.81E-02					CXCR2
NR5A2		ligand-dependent nuclear receptor	2.01E-02	6.81E-02					CEBPD,GOT1,IL1RN
GRK4		kinase	2.16E-02	6.81E-02					AGTRAP
PLAA		other	2.16E-02	6.81E-02					PTGS2
Cxcl3		cytokine	2.16E-02	6.81E-02					CEBPD
KMT2B		transcription regulator	2.16E-02	6.81E-02					IRAK3
CD28		transmembrane receptor	1.99E-02	6.81E-02		-1.172	bias	-2.184	BTG1,GNL2,PTGS2,RPL6,RPS14
fluticasone		chemical drug	2.16E-02	6.81E-02					TLR4
2-cyclohexen-1-one		chemical - endogenous non-mammalian	2.16E-02	6.81E-02					PTGS2
TO-901317		chemical reagent	2.05E-02	6.81E-02					ACSL1,PFKFB3,PTGS2,TLR4
pyridoxal phosphate-6-azophenyl-2',4'-disulfonic acid		chemical reagent	2.16E-02	6.81E-02					PTGS2
phenylmethylsulfonyl fluoride		chemical - protease inhibitor	2.16E-02	6.81E-02					TLR4
advanced glycation end-products		chemical - endogenous mammalian	2.17E-02	6.81E-02					PTGS2,TLR4
cytochalasin D		chemical toxicant	2.01E-02	6.81E-02					CXCR2,PTGS2
mevalonolactone		chemical reagent	2.16E-02	6.81E-02					PTGS2
atrasentan		chemical drug	2.16E-02	6.81E-02					PTGS2
bicalutamide		chemical drug	2.01E-02	6.81E-02					F5,FKBP5
lansoprazole		chemical drug	2.16E-02	6.81E-02					PTGS2
butylated hydroxytoluene		chemical toxicant	2.16E-02	6.81E-02					PTGS2
semaxinib		chemical drug	2.13E-02	6.81E-02					IL18RAP,PARP1,PTGS2
vanadyl sulfate		chemical reagent	2.16E-02	6.81E-02					IL1RN
1-docosapentaenoylglycerol		chemical reagent	2.16E-02	6.81E-02					PTGS2
sappanone A		chemical - endogenous non-mammalian	2.16E-02	6.81E-02					PTGS2
Phe-Pro-Arg-chloromethyl ketone		chemical - protease inhibitor	2.16E-02	6.81E-02					OSM

cockroach extract		chemical reagent	2.16E-02	6.81E-02					PTGS2
aclarubicin		chemical drug	2.16E-02	6.81E-02					PTGS2
chlorine		chemical toxicant	2.16E-02	6.81E-02					ALOX5
NADPH		chemical - endogenous mammalian	2.16E-02	6.81E-02					PTGS2
5-hydroxytryptophan		chemical - endogenous mammalian	2.16E-02	6.81E-02					PTGS2
carrageenan		chemical drug	2.17E-02	6.81E-02					PARP1,PTGS2
Cr6+		chemical reagent	2.16E-02	6.81E-02					SLC30A1
MET		kinase	2.18E-02	6.82E-02					CXCL1,NFKBIZ,PTGS2
ERBB2		kinase	2.23E-02	6.97E-02		0.688	bias	0.03	BCL3,ETV6,LAMP2,MME,PFKFB3,PTGS2,PTTG1IP,RNF149,STEAP4
nitroprusside		chemical drug	2.25E-02	7.03E-02					PARP1,PTGS2
EGFR		kinase	2.29E-02	7.15E-02					BTG1,MME,PTGS2,PTTG1IP,SLC2A3,STEAP4
mibolerone		chemical drug	2.30E-02	7.18E-02					ACSL1,FKBP5,MME
tetrodotoxin		chemical drug	2.39E-02	7.41E-02					LRRK2,NSUN7,PTGS2
SOCS1		other	2.39E-02	7.41E-02					FKBP5,MYD88,PTGS2
GNAQ		enzyme	2.39E-02	7.41E-02					IL1RN,PRKCD,PTGS2
ciglitazone		chemical drug	2.39E-02	7.41E-02					CXCL1,PARP1,PTGS2
FGF10		growth factor	2.41E-02	7.47E-02					NUMB,PTGS2
TET2		enzyme	2.43E-02	7.51E-02					BCL3,FBXL5,HAL
PPARG		ligand-dependent nuclear receptor	2.43E-02	7.51E-02		0.272		-0.179	ACSL1,CXCL1,MYD88,PFKFB3,PTGS2,TLR4
tacrolimus		chemical drug	2.43E-02	7.51E-02		-1.969		-1.487	F5,FKBP5,PTGS2,SELL
3'-O-(4-benzoyl)benzoyladenosine 5'-triphosphate		chemical reagent	2.59E-02	7.52E-02					PTGS2
GPIIB-IIIA		complex	2.59E-02	7.52E-02					BCL3
GTP		chemical - endogenous mammalian	2.59E-02	7.52E-02					GNL3
lenalidomide		chemical drug	2.56E-02	7.52E-02		0.655		0.334	NFKBIZ,SPI1,TLR4,WDFY3
teichoic acid		chemical - endogenous non-mammalian	2.59E-02	7.52E-02					PTGS2
P110		group	2.59E-02	7.52E-02					PRKCD
lupeol		chemical drug	2.59E-02	7.52E-02					PTGS2
5R-hydroxytriptolide		chemical drug	2.59E-02	7.52E-02					TLR4
LRRC26		ion channel	2.59E-02	7.52E-02					CXCL1
RAB7B		peptidase	2.59E-02	7.52E-02					TLR4
CCAR1		transcription regulator	2.59E-02	7.52E-02					CEBPD
NLRP10		enzyme	2.59E-02	7.52E-02					ALOX5
RNF152		enzyme	2.59E-02	7.52E-02					CXCL1
estrogen receptor		group	2.59E-02	7.52E-02		-1		-0.979	CXCL1,LYN,SELL,TLR4
TNFRSF10A		transmembrane receptor	2.59E-02	7.52E-02					ITGAM
FES		kinase	2.59E-02	7.52E-02					ITGAM
miR-219a-5p (and other miRNAs w/seed GAUUGUC)		mature microRNA	2.59E-02	7.52E-02					ALOX5
NUCB2		other	2.59E-02	7.52E-02					PTGS2
LTA		cytokine	2.57E-02	7.52E-02					PTGS2,SELL
ADAR		enzyme	2.59E-02	7.52E-02					SPI1
resolvin D5		chemical - endogenous mammalian	2.59E-02	7.52E-02					ITGAM
ORM1		other	2.59E-02	7.52E-02					IL1RN
SSPN		other	2.59E-02	7.52E-02					PTGS2
PRSS8		peptidase	2.59E-02	7.52E-02					TLR4
TAB2		other	2.59E-02	7.52E-02					PTGS2
ACSL4		enzyme	2.49E-02	7.52E-02					PTGS2,SELL
RGFP966		chemical reagent	2.59E-02	7.52E-02					MME
UBE2D1		enzyme	2.59E-02	7.52E-02					PTGS2
glaucocalyxin A		chemical - endogenous non-mammalian	2.59E-02	7.52E-02					PTGS2
GABPA		transcription regulator	2.57E-02	7.52E-02					FCAR,SPI1
CBL		transcription regulator	2.49E-02	7.52E-02					IL17RA,LYN
WRN		enzyme	2.59E-02	7.52E-02					PRKCD
MATK		kinase	2.59E-02	7.52E-02					LYN
CLEC11A		growth factor	2.49E-02	7.52E-02					ALOX5,SPI1
CD5		transmembrane receptor	2.57E-02	7.52E-02					BCL3,TLR8
ANP32A		other	2.59E-02	7.52E-02					PTGS2
MIA-459		chemical reagent	2.59E-02	7.52E-02					PTGS2
MIA-313		chemical reagent	2.59E-02	7.52E-02					PTGS2
gelam honey		chemical reagent	2.59E-02	7.52E-02					PTGS2
furosemide		chemical drug	2.59E-02	7.52E-02					PTGS2
EGTA acetoxymethyl ester		chemical reagent	2.59E-02	7.52E-02					PTGS2
olmesartan medoxomil		chemical drug	2.59E-02	7.52E-02					PTGS2
ferric nitrilotriacetate		chemical toxicant	2.59E-02	7.52E-02					PTGS2

mibefradil		chemical drug	2.59E-02	7.52E-02					SELL
dibenzoylmethane		chemical toxicant	2.59E-02	7.52E-02					PTGS2
glucagon		biologic drug	2.57E-02	7.52E-02					MME,PLXDC2
Z-VRPR-FMK		chemical - protease inhibitor	2.59E-02	7.52E-02					LAMP2
propyl gallate		chemical toxicant	2.59E-02	7.52E-02					PTGS2
ZFA-fmk		chemical reagent	2.59E-02	7.52E-02					LAMP2
ingenol mebutate		chemical drug	2.59E-02	7.52E-02					PRKCD
luteolin		chemical drug	2.57E-02	7.52E-02					PTGS2,TLR4
ginsenoside Rg1		chemical - endogenous non-mammalian	2.59E-02	7.52E-02					PTGS2
ciprofibrate		chemical drug	2.61E-02	7.58E-02					GOT1,HAL,QPCT
testosterone		chemical - endogenous mammalian	2.63E-02	7.61E-02		0.849	bias	0.225	ACSL1,BCL3,PARP1,PTGS2,TLR4
PIM1		kinase	2.66E-02	7.65E-02					GNL3,SELL
CD14		transmembrane receptor	2.66E-02	7.65E-02					CXCR2,PTGS2
GNRH1		other	2.66E-02	7.65E-02					PRKCD,PTGS2
NR3C2		ligand-dependent nuclear receptor	2.66E-02	7.65E-02					CXCR2,FKBP5,PTGS2
di(2-ethylhexyl) phthalate		chemical toxicant	2.66E-02	7.65E-02					CXCL1,MYD88
morphine		chemical drug	2.66E-02	7.65E-02					ITGAM,PTGS2,TLR4
PRDM1		transcription regulator	2.69E-02	7.71E-02		0		0.265	F5,HAL,IL18RAP,SELL
Akt		group	2.71E-02	7.76E-02		1.189		0.689	FKBP5,MME,PTGS2,PTTG1IP,STEAP4
palmitic acid		chemical - endogenous mammalian	2.72E-02	7.79E-02		1.959	bias	1.428	ACSL1,IL1RN,PTGS2,TLR4
Vegf		group	2.72E-02	7.80E-02	Activated	2.219	bias		CXCL1,CXCR2,FKBP5,LYN,NPL,PTGS2
NCOR2		transcription regulator	2.74E-02	7.80E-02					OSM,PTGS2
HSP90B1		other	2.74E-02	7.80E-02					RPS14,TLR4
RETN		other	2.74E-02	7.80E-02					PTGS2,TLR4
PRKCB		kinase	2.74E-02	7.80E-02					PRKCD,PTGS2
ZAP70		kinase	2.74E-02	7.80E-02					FKBP5,QPCT
telmisartan		chemical drug	2.74E-02	7.80E-02					ACSL1,PTGS2
decitabine		chemical drug	2.75E-02	7.80E-02		1.245	bias	0.507	ALOX5,BTG1,PTGS2,RPL4,SELL,SLC2A3,SLC30A1,SPI1
troglitazone		chemical drug	2.76E-02	7.83E-02		-1.686		-1.795	ACSL1,CEBPD,CXCL1,MME,PTGS2
CSF1		cytokine	2.79E-02	7.89E-02		1.091	bias	0.129	ITGAM,PFKFB3,SPI1,TLR1
adenosine		chemical - endogenous mammalian	2.83E-02	7.98E-02					PTGS2,TLR4
PPP2CA		phosphatase	2.83E-02	7.98E-02					PTGS2,SELL
IL1R1		transmembrane receptor	2.83E-02	7.98E-02					CXCL1,PTGS2
PTGES		enzyme	2.83E-02	7.98E-02					LAMP2,PTGS2
Tnf (family)		group	2.85E-02	8.02E-02					CXCL1,PTGS2,SELL
BRD4		kinase	2.85E-02	8.02E-02					BCL3,ETV6,MYD88
EZH2		transcription regulator	2.87E-02	8.06E-02					CXCL1,FKBP5,IL18RAP,IRAK3,PTGS2
hydrocortisone		chemical - endogenous mammalian	2.99E-02	8.08E-02					CEBPD,ITGAM,PTGS2
(6)-gingerol		chemical - endogenous non-mammalian	3.01E-02	8.08E-02					PTGS2
Smad1/5/8-Smad4		complex	3.01E-02	8.08E-02					PTGS2
BCAR4		other	3.01E-02	8.08E-02					PFKFB3
NOX5		ion channel	3.01E-02	8.08E-02					PTGS2
QRFP		other	3.01E-02	8.08E-02					ACSL1
sphingomyelinase		group	3.01E-02	8.08E-02					PTGS2
urotensin II		biologic drug	3.01E-02	8.08E-02					ALOX5
PDGFD		growth factor	3.01E-02	8.08E-02					PTGS2
AZD4547		chemical drug	3.01E-02	8.08E-02					CXCL1
DVL1		other	3.01E-02	8.08E-02					PTGS2
ENO1		enzyme	3.01E-02	8.08E-02					PTGS2
miR-511-5p (miRNAs w/seed UGUCUUU)		mature microRNA	3.01E-02	8.08E-02					TLR4
miR-542-3p (miRNAs w/seed GUGACAG)		mature microRNA	3.01E-02	8.08E-02					PTGS2
SWAP70		other	3.01E-02	8.08E-02					ITGAM
TDO2		enzyme	3.01E-02	8.08E-02					PTGS2
PPM1B		phosphatase	3.01E-02	8.08E-02					CEBPD
TPSD1		peptidase	3.01E-02	8.08E-02					PTGS2
TBXA2R		G-protein coupled receptor	3.01E-02	8.08E-02					PTGS2
PROK1		growth factor	3.01E-02	8.08E-02					PTGS2
HNRNPUL1		other	3.01E-02	8.08E-02					PARP1
LPAR2		G-protein coupled receptor	3.01E-02	8.08E-02					PTGS2
APOBEC1		enzyme	3.01E-02	8.08E-02					PTGS2
IL18RAP	0.42	transmembrane receptor	3.01E-02	8.08E-02					PTGS2
PLA2G2A		enzyme	3.01E-02	8.08E-02					PTGS2
RXRG		ligand-dependent nuclear receptor	2.92E-02	8.08E-02					ACSL1,OSM
XPA		other	3.01E-02	8.08E-02					PTGS2

HRAS		enzyme	2.91E-02	8.08E-02		1.202		0.775	CEBPD,CXCL1,ITGAM,OSM,PRKCD,PTGS2,RPS3
PITPNA		transporter	3.01E-02	8.08E-02					PTGS2
QKI		other	2.92E-02	8.08E-02					ITGAM,LAMP2
Pde4d		enzyme	3.01E-02	8.08E-02					PTGS2
NKRF		transcription regulator	3.01E-02	8.08E-02					PTGS2
4-coumaric acid		chemical - endogenous mammalian	3.01E-02	8.08E-02					PTGS2
10-(6'-ubiquinonyl)decyltriphenylphosphonium		chemical drug	3.01E-02	8.08E-02					PTGS2
carbon monoxide		chemical - endogenous mammalian	3.01E-02	8.08E-02					PTGS2,TLR4
RWJ 67657		chemical - kinase inhibitor	3.01E-02	8.08E-02					PTGS2
Ki16425		chemical reagent	3.01E-02	8.08E-02					PTGS2
eugenol		chemical - endogenous non-mammalian	3.01E-02	8.08E-02					PTGS2
rutin		chemical drug	3.01E-02	8.08E-02					PTGS2
atropine		chemical drug	3.01E-02	8.08E-02					PTGS2
phosphatidic acid		chemical - endogenous mammalian	3.01E-02	8.08E-02					PTGS2
12-hydroxyeicosatetraenoic acid		chemical - endogenous mammalian	3.01E-02	8.08E-02					PTGS2
S-allyl-L-cysteine		chemical - endogenous non-mammalian	3.01E-02	8.08E-02					PTGS2
L-triiodothyronine		chemical - endogenous mammalian	3.07E-02	8.22E-02		0.298	bias	-0.345	ACSL1,AGTRAP,BCL3,PARP1,PTGS2,RPS14
OSMR		transmembrane receptor	3.10E-02	8.28E-02					BCL3,OSM
CXCR4		G-protein coupled receptor	3.10E-02	8.28E-02					CXCL1,CXCR2
CD40LG		cytokine	3.12E-02	8.32E-02		1.199	bias		BTG1,CXCL1,ITGAM,PTGS2,SELL
17-alpha-ethinylestradiol		chemical drug	3.15E-02	8.38E-02					BCL3,CXCR2,IL17RA
PTGER4		G-protein coupled receptor	3.15E-02	8.38E-02					OSM,PTGS2,TLR8
BSCL2		other	3.28E-02	8.72E-02					ACSL1,PTGS2
glucosamine		chemical - endogenous mammalian	3.28E-02	8.72E-02					CXCL1,PTGS2
10E,12Z-octadecadienoic acid		chemical - endogenous mammalian	3.30E-02	8.76E-02					CREB5,IL1RN,PTGS2
12(S)-hydroxyeicosatetraenoic acid		chemical - endogenous non-mammalian	3.44E-02	8.83E-02					PTGS2
ganglioside GD1a		chemical - endogenous mammalian	3.44E-02	8.83E-02					PTGS2
ganglioside GM1		chemical - endogenous mammalian	3.44E-02	8.83E-02					PTGS2
infliximab		biologic drug	3.37E-02	8.83E-02					CXCL1,PTGS2
coconut oil		chemical drug	3.44E-02	8.83E-02					PTGS2
cyanidin 3-O-glucoside		chemical - endogenous non-mammalian	3.44E-02	8.83E-02					PTGS2
PPT1		enzyme	3.44E-02	8.83E-02					OSM
CACNA1C		ion channel	3.44E-02	8.83E-02					PTGS2
PON2		enzyme	3.44E-02	8.83E-02					CXCR2
ADGRF5		G-protein coupled receptor	3.44E-02	8.83E-02					IL1RN
immune complex		complex	3.44E-02	8.83E-02					IL1RN
CD82		other	3.44E-02	8.83E-02					LYN
CD5L		transmembrane receptor	3.44E-02	8.83E-02					TLR4
EGLN1		enzyme	3.37E-02	8.83E-02					PTGS2,SELL
HPGDS		enzyme	3.44E-02	8.83E-02					ITGAM
GRIP1		transcription regulator	3.44E-02	8.83E-02					PTGS2
ZNF711		transcription regulator	3.44E-02	8.83E-02					OSM
FYB1		other	3.44E-02	8.83E-02					ITGAM
PLA2G5		enzyme	3.44E-02	8.83E-02					PTGS2
CCL-34		chemical reagent	3.44E-02	8.83E-02					ITGAM
naproxen		chemical drug	3.44E-02	8.83E-02					PTGS2
NCX-4040		chemical drug	3.44E-02	8.83E-02					PTGS2
GW 5074		chemical - kinase inhibitor	3.44E-02	8.83E-02					PTGS2
benzoic acid		chemical - endogenous mammalian	3.44E-02	8.83E-02					ITGAM
domoic acid		chemical toxicant	3.44E-02	8.83E-02					PTGS2
carnosol		chemical - endogenous non-mammalian	3.44E-02	8.83E-02					PTGS2
ST1926		chemical drug	3.40E-02	8.83E-02					FKBP5,RPL6,TOMM7
JNJ-39933673		chemical reagent	3.44E-02	8.83E-02					TLR4
cinnamon powder		chemical reagent	3.44E-02	8.83E-02					SLC2A3
palmitoyl-Cys((RS)-2,3-di(palmitoyloxy)-propyl)-Ala-Gly-OH		chemical reagent	3.44E-02	8.83E-02					PTGS2
sulfo-N-succinimidyl oleate		chemical toxicant	3.44E-02	8.83E-02					TLR4

tosyllysine chloromethyl ketone		chemical - protease inhibitor	3.44E-02	8.83E-02					PTGS2
diphenyleneiodonium		chemical reagent	3.47E-02	8.89E-02					PTGS2,SELL
Go 6976		chemical - kinase inhibitor	3.47E-02	8.89E-02					LAMP2,PTGS2
UCP1		transporter	3.51E-02	8.99E-02					GOT1,SHMT2,SPI1
vorinostat		chemical drug	3.55E-02	9.08E-02		-1.429		-1.493	OSM,PARP1,PTGS2,SPI1
APC		enzyme	3.57E-02	9.09E-02					CXCR2,ITGAM,PTGS2
trovafloxacin		chemical drug	3.56E-02	9.09E-02					IL17RA,MYD88
folic acid		chemical - endogenous mammalian	3.56E-02	9.09E-02					MYD88,NUMB
dextran sulfate		chemical drug	3.59E-02	9.14E-02					CXCR2,PARP1,PTGS2,TLR4
PRKAG3		other	3.62E-02	9.21E-02					GOT1,RPL5,SLC2A3
DOCK8		other	3.66E-02	9.26E-02					NFKBIZ,PTGS2
PDGFB		growth factor	3.66E-02	9.26E-02					IL1RN,PTGS2
TBX21		transcription regulator	3.66E-02	9.26E-02					IL18RAP,SELL
caffeine		chemical drug	3.66E-02	9.26E-02					ITGAM,PTGS2
25-hydroxycholesterol		chemical reagent	3.66E-02	9.26E-02					ACSL1,PTGS2
ganglioside		chemical - endogenous mammalian	3.86E-02	9.27E-02					PTGS2
2',3'-dialdehyde ATP		chemical reagent	3.86E-02	9.27E-02					PTGS2
tannic acid		chemical toxicant	3.86E-02	9.27E-02					PTGS2
RUNX1T1		transcription regulator	3.86E-02	9.27E-02					SPI1
abatacept		biologic drug	3.86E-02	9.27E-02					SELL
motexafin gadolinium		chemical drug	3.86E-02	9.27E-02					SLC30A1
Agtr1b		G-protein coupled receptor	3.86E-02	9.27E-02					PTGS2
atrazine		chemical toxicant	3.86E-02	9.27E-02					PTGS2
S6K1		group	3.86E-02	9.27E-02					BTG1
APH1A		peptidase	3.86E-02	9.27E-02					NCSTN
bacterial lipopolysaccharides		chemical - other	3.86E-02	9.27E-02					BCL3
BCG vaccine		biologic drug	3.86E-02	9.27E-02					PTGS2
SLC16A3		transporter	3.86E-02	9.27E-02					PFKFB3
FCGR3A/FCGR3B		transmembrane receptor	3.86E-02	9.27E-02					SELL
RSPO3		kinase	3.86E-02	9.27E-02					PTGS2
PHF8		enzyme	3.86E-02	9.27E-02					IRAK3
salmonella typhimurium lipopolysaccharide		chemical - endogenous non-mammalian	3.86E-02	9.27E-02					PTGS2
TACSTD2		other	3.86E-02	9.27E-02					PARP1
UCHL5		peptidase	3.86E-02	9.27E-02					PARP1
NEK7		kinase	3.86E-02	9.27E-02					PTGS2
dynasore		chemical reagent	3.86E-02	9.27E-02					PTGS2
idelalisib		chemical drug	3.86E-02	9.27E-02					SELL
TRG		other	3.86E-02	9.27E-02					CXCL1
PSMB9		peptidase	3.86E-02	9.27E-02					RPS3
S100a7a		other	3.86E-02	9.27E-02					CXCL1
RAB1A		enzyme	3.86E-02	9.27E-02					PRKCD
GUSB		enzyme	3.86E-02	9.27E-02					CEBPD
ZEB2		transcription regulator	3.76E-02	9.27E-02					CYP4F3,SELL
IL4I1		enzyme	3.86E-02	9.27E-02					CXCL1
UGT2B17		enzyme	3.86E-02	9.27E-02					FKBP5
SMARCA2		transcription regulator	3.76E-02	9.27E-02					FKBP5,ITGAM
NEK6		kinase	3.86E-02	9.27E-02					PTGS2
ITGB3		transmembrane receptor	3.76E-02	9.27E-02					OSM,PTGS2
SASH1		other	3.85E-02	9.27E-02					NFKBIZ,PTGS2
UACA		other	3.86E-02	9.27E-02					CEBPD
PPP2R1A		phosphatase	3.86E-02	9.27E-02					FPR2
ANPEP		peptidase	3.86E-02	9.27E-02					IL1RN
GCM1		transcription regulator	3.86E-02	9.27E-02					OSM
UGT2B15		enzyme	3.86E-02	9.27E-02					FKBP5
AP3B1		transporter	3.86E-02	9.27E-02					LAMP2
Map3k7		kinase	3.76E-02	9.27E-02					NFKBIZ,PTGS2
geldanamycin		chemical drug	3.71E-02	9.27E-02	Inhibited	-2		-1.583	CEBPD,FKBP5,PARP1,PTGS2
cyclopentenone		chemical reagent	3.86E-02	9.27E-02					PTGS2
racemic flurbiprofen		chemical drug	3.86E-02	9.27E-02					PTGS2
7,8-dihydro-7,8-dihydroxybenzo(a)pyrene 9,10-oxide		chemical toxicant	3.86E-02	9.27E-02					PTGS2
ketorolac		chemical drug	3.86E-02	9.27E-02					PTGS2
retinaldehyde		chemical - endogenous mammalian	3.86E-02	9.27E-02					PTGS2
JMF3086		chemical reagent	3.86E-02	9.27E-02					PTGS2
glutathione		chemical - endogenous mammalian	3.76E-02	9.27E-02					GNL3,PTGS2
hexamethoxyflavone		chemical toxicant	3.86E-02	9.27E-02					PTGS2
I-BOP		chemical reagent	3.86E-02	9.27E-02					PTGS2

saturated fatty acid		chemical - other	3.86E-02	9.27E-02					PTGS2
arachidonyltrifluoromethane		chemical reagent	3.86E-02	9.27E-02					PTGS2
miricorilant		chemical reagent	3.86E-02	9.27E-02					FKBP5
MRTFB		transcription regulator	3.96E-02	9.45E-02					ETV6,NFKBIZ,PTGS2
TEAD1		transcription regulator	3.95E-02	9.45E-02					NFKBIZ,PTGS2
GNA14		enzyme	3.95E-02	9.45E-02					IL1RN,PTGS2
FBXW7		enzyme	3.95E-02	9.45E-02					CEBPD,TLR4
FLT3LG		cytokine	3.95E-02	9.45E-02					ITGAM,SPI1
SHC1		other	4.05E-02	9.68E-02					CXCL1,ITGAM
CREB1		transcription regulator	4.06E-02	9.69E-02		1.251		0.766	ATP6V0B,CEBPD,ETV6,PLXDC2,PTGS2,SLC2A3
PTH		other	4.13E-02	9.84E-02					MME,PLXDC2,PTGS2
corticosteroid		chemical drug	4.26E-02	9.86E-02					FKBP5,IL1RN
voltage-gated calcium channel		complex	4.28E-02	9.86E-02					ITGAM
PI3KÎ³		group	4.28E-02	9.86E-02					TLR4
F630028O10Rik		other	4.28E-02	9.86E-02					ITGAM
NQO2		enzyme	4.28E-02	9.86E-02					PTGS2
B4GALT6		enzyme	4.28E-02	9.86E-02					PTGS2
PHLPP2		enzyme	4.28E-02	9.86E-02					PRKCD
LGALS8		other	4.28E-02	9.86E-02					CXCL1
CXCL5		cytokine	4.28E-02	9.86E-02					ITGAM
LINC01234		other	4.28E-02	9.86E-02					SHMT2
KRT19		other	4.28E-02	9.86E-02					NUMB
ITGA4		transmembrane receptor	4.28E-02	9.86E-02					IL1RN
mir-506		microRNA	4.28E-02	9.86E-02					TLR4
mir-634		microRNA	4.28E-02	9.86E-02					LAMP2
PIK3CA		kinase	4.26E-02	9.86E-02					CEBPD,ITGAM
NOD2		other	4.26E-02	9.86E-02					ACSL1,ITGAM
ADIPOR2		transmembrane receptor	4.28E-02	9.86E-02					PTGS2
RNF41		enzyme	4.28E-02	9.86E-02					MYD88
HOXB7		transcription regulator	4.28E-02	9.86E-02					CXCL1
GPC1		transmembrane receptor	4.28E-02	9.86E-02					PTGS2
CDKN1C		other	4.28E-02	9.86E-02					PARP1
Brd4		kinase	4.15E-02	9.86E-02					TLR4,TLR8
mito-TEMPO		chemical reagent	4.28E-02	9.86E-02					PTGS2
BP-1-102		chemical reagent	4.28E-02	9.86E-02					IL1RN
allyl isothiocyanate		chemical toxicant	4.28E-02	9.86E-02					PTGS2
calmidazolium		chemical drug	4.28E-02	9.86E-02					SELL
trifluoperazine		chemical drug	4.28E-02	9.86E-02					SELL
clomipramine		chemical drug	4.28E-02	9.86E-02					SLC2A3
(5-(4-N-methyl-N(2-pyridyl)amino)ethoxy)benzyl thiazolidine-2,4-dione		chemical reagent	4.28E-02	9.86E-02					ITGAM
cortistatin A		chemical reagent	4.28E-02	9.86E-02					ETV6
TAPI-1		chemical - protease inhibitor	4.28E-02	9.86E-02					CXCR2
gossypol		chemical drug	4.28E-02	9.86E-02					CXCL1
tyrphostin AG 1024		chemical - kinase inhibitor	4.28E-02	9.86E-02					PTGS2
C-miR146a		chemical reagent	4.28E-02	9.86E-02					MYD88
AI-1		chemical reagent	4.28E-02	9.86E-02					GOT1
cadmium		chemical toxicant	4.26E-02	9.86E-02					PTGS2,SLC30A1
trichostatin A		chemical drug	4.32E-02	9.95E-02		-0.128		-0.581	BTG1,ITGAM,LAGE3,MME,OSM,PRKCD,PTGS2,TLR4
Il3		cytokine	4.36E-02	1.00E-01					ITGAM,OSM
PF4		cytokine	4.36E-02	1.00E-01					ITGAM,NFKBIZ
CALCA		other	4.37E-02	1.00E-01					MME,PLXDC2,SPI1
ESR1		ligand-dependent nuclear receptor	4.45E-02	1.02E-01		0		-0.285	ACSL1,ALOX5,CEBPD,CXCL1,IL17RA,LAPTM5,MME,PRKCD,PTGS2,PTTG1IP,SELL
ursolic acid		chemical drug	4.46E-02	1.02E-01					PTGS2,TLR4
PPARGC1A		transcription regulator	4.47E-02	1.02E-01		-1.715		-1.978	CEBPD,IL1RN,MYD88,NFKBIZ,PTGS2
lysophosphatidylcholine		chemical - other	4.57E-02	1.03E-01					CXCL1,PTGS2
triciribine		chemical drug	4.70E-02	1.03E-01					PARP1
pirinixic acid		chemical toxicant	4.65E-02	1.03E-01		1.292	bias	0.327	ACSL1,GOT1,IL1RN,ITGAM,PTGS2
1L-6-hydroxymethyl-chiro-inositol 2-(R)-2-O-methyl-3-O-octadecylcarbonate		chemical - kinase inhibitor	4.70E-02	1.03E-01					PTGS2
ganglioside GT1		chemical - endogenous mammalian	4.70E-02	1.03E-01					PTGS2
EPHB1		kinase	4.70E-02	1.03E-01					PTGS2
1-hydroxy-2-methyl-2-butenyl 4-diphosphate		chemical - endogenous non-mammalian	4.70E-02	1.03E-01					OSM
ERK1/2		group	4.55E-02	1.03E-01					ALOX5,CXCL1,FKBP5,PTGS2
coal tar		chemical drug	4.70E-02	1.03E-01					PTGS2
MSI2		other	4.70E-02	1.03E-01					NUMB
APOL1		transporter	4.70E-02	1.03E-01					TLR1
T 0070907		chemical reagent	4.70E-02	1.03E-01					PTGS2

MTF1		transcription regulator	4.70E-02	1.03E-01					SLC30A1
astragalin		chemical - endogenous non-mammalian	4.70E-02	1.03E-01					PTGS2
docosapentaenoic acid		chemical reagent	4.70E-02	1.03E-01					PTGS2
PLCB1		enzyme	4.70E-02	1.03E-01					PTGS2
LNX2		other	4.70E-02	1.03E-01					NUMB
LEPR		transmembrane receptor	4.67E-02	1.03E-01					BTG1,IL1RN,ITGAM
KLF7		transcription regulator	4.70E-02	1.03E-01					IL1RN
MAPK7		kinase	4.57E-02	1.03E-01					PTGS2,STEAP4
CNB-001		chemical reagent	4.70E-02	1.03E-01					ALOX5
JMJD1C		enzyme	4.70E-02	1.03E-01					ETV6
NDFIP1		other	4.70E-02	1.03E-01					LYN
PPBP		cytokine	4.70E-02	1.03E-01					CXCR2
SREBF2		transcription regulator	4.67E-02	1.03E-01					ACSL1,CXCL1
ELF2		transcription regulator	4.70E-02	1.03E-01					LYN
S100A7		other	4.70E-02	1.03E-01					CXCL1
J11-Cl		chemical reagent	4.70E-02	1.03E-01					PTGS2
sesame oil		chemical reagent	4.70E-02	1.03E-01					PTGS2
2-aminoethoxydiphenylborane		chemical reagent	4.70E-02	1.03E-01					PTGS2
zileuton		chemical drug	4.70E-02	1.03E-01					PTGS2
sesamin		chemical - endogenous non-mammalian	4.70E-02	1.03E-01					PTGS2
manumycin A		chemical reagent	4.70E-02	1.03E-01					PTGS2
lidocaine		chemical drug	4.70E-02	1.03E-01					PARP1
captopril		chemical drug	4.67E-02	1.03E-01					IL1RN,PTGS2
SD6		chemical reagent	4.70E-02	1.03E-01					CXCL1
kukoamine A		chemical - endogenous non-mammalian	4.70E-02	1.03E-01					ALOX5
enterolactone		chemical - endogenous mammalian	4.70E-02	1.03E-01					IL1RN
rosmarinic acid		chemical - endogenous non-mammalian	4.70E-02	1.03E-01					PTGS2
CA074-methyl ester		chemical reagent	4.70E-02	1.03E-01					LAMP2
forskolin		chemical toxicant	4.52E-02	1.03E-01	Activated	2.55	bias	1.698	ATP6V0B,BCL3,CEBPD,CXCL1,FKBP5,PTGS2,VNN2
puerarin		chemical drug	4.70E-02	1.03E-01					PTGS2
paclitaxel		chemical drug	4.55E-02	1.03E-01					CXCL1,CYP4F3,PTGS2,SLC2A3
allopregnanolone		chemical - endogenous mammalian	4.70E-02	1.03E-01					PTGS2
nandrolone		chemical drug	4.70E-02	1.03E-01					NUMB
diethylstilbestrol		chemical drug	4.74E-02	1.04E-01		0.686	bias	0.132	IGSF6,LRRK2,PTTG1IP,TSHZ3
enterotoxin B		biologic drug	4.78E-02	1.05E-01					CXCL1,MYD88
KLF6		transcription regulator	4.78E-02	1.05E-01					ALOX5,PTGS2
GSKJ4		chemical reagent	4.78E-02	1.05E-01					PFKFB3,SLC2A3
N-Ac-Leu-Leu-norleucinal		chemical - protease inhibitor	4.78E-02	1.05E-01					LAMP2,PTGS2
IGF1		growth factor	4.79E-02	1.05E-01		1.274	bias	0.514	CEBPD,ETV6,PARP1,PLXDC2,PTGS2,TLR4
KLF2		transcription regulator	4.86E-02	1.06E-01					BCL3,PTGS2,SELL
epinephrine		chemical - endogenous mammalian	4.89E-02	1.07E-01					PTGS2,SELL
PTGS2	0.33	enzyme	4.93E-02	1.07E-01					CXCR2,PTGS2,SELL
butyric acid		chemical - endogenous mammalian	4.95E-02	1.08E-01		-0.117		-0.226	ALOX5,ITGAM,PDK3,PRKCD,PTGS2
IFN alpha/beta		group	4.99E-02	1.08E-01					CCRL2,TLR1
AIRE		transcription regulator	4.99E-02	1.08E-01					TLR1,TLR8
TGFA		growth factor	4.99E-02	1.08E-01					ITGAM,PTGS2
C3		peptidase	4.99E-02	1.08E-01					ITGAM,SELL
PPP3CA		phosphatase	4.99E-02	1.08E-01					LAMP2,PTGS2
ESRRA		transcription regulator	4.99E-02	1.08E-01					ACSL1,GOT1,PRKCD
MYCN		transcription regulator	5.91E-02	1.16E-01	Inhibited	-2		-2.271	RPL4,RPL5,RPL6,RPS3
5-fluorouracil		chemical drug	6.61E-02	1.24E-01	Activated	2		2.341	PTGS2,RPL5,RPL6,TLR4
doxorubicin		chemical drug	1.34E-01	1.90E-01		1.953		1.472	BTG1,CXCL1,PTGS2,TLR4
