# Supplementary material for: Preimmunization correlates of protection shared across malaria vaccine trials in adults
Source: NPJ Vaccines. 2022 Jan 14;7:5. doi: 10.1038/s41541-021-00425-1 (PMC8760258; doi:10.1038/s41541-021-00425-1)
Supplement: Supplementary file 2 — REPORTING SUMMARY [file 41541_2021_425_MOESM2_ESM.pdf]

nature portfolio

Corresponding author(s): Stuart, Aitchison

Last updated by author(s): Oct 8, 2021

Reporting Summary

Nature Portfolio wishes to improve the reproducibility of the work that we publish. This form provides structure for consistency and transparency in reporting. For further information on Nature Portfolio policies, see our Editorial Policies and the Editorial Policy Checklist.

Statistics

For all statistical analyses, confirm that the following items are present in the figure legend, table legend, main text, or Methods section.

n/a	Confirmed	
☐	☒	The exact sample size (*n*) for each experimental group/condition, given as a discrete number and unit of measurement
☐	☒	A statement on whether measurements were taken from distinct samples or whether the same sample was measured repeatedly
☐	☒	The statistical test(s) used AND whether they are one- or two-sided<br>*Only common tests should be described solely by name; describe more complex techniques in the Methods section.*
☒	☐	A description of all covariates tested
☐	☒	A description of any assumptions or corrections, such as tests of normality and adjustment for multiple comparisons
☐	☒	A full description of the statistical parameters including central tendency (e.g. means) or other basic estimates (e.g. regression coefficient) AND variation (e.g. standard deviation) or associated estimates of uncertainty (e.g. confidence intervals)
☒	☐	For null hypothesis testing, the test statistic (e.g. *F*, *t*, *r*) with confidence intervals, effect sizes, degrees of freedom and *P* value noted<br>*Give P values as exact values whenever suitable.*
☒	☐	For Bayesian analysis, information on the choice of priors and Markov chain Monte Carlo settings
☒	☐	For hierarchical and complex designs, identification of the appropriate level for tests and full reporting of outcomes
☒	☐	Estimates of effect sizes (e.g. Cohen's *d*, Pearson's *r*), indicating how they were calculated

Our web collection on statistics for biologists contains articles on many of the points above.

Software and code

Policy information about availability of computer code

Data collection

The study analyzed data that had been collected previously. We did not collect new data as part of this research.

Data analysis

Raw RNA-seq sequencing data were aligned to the hg19 human reference genome using a previously described pipeline (PMID: 29050771). Briefly, read pairs were adjusted to set base calls with phred scores less than 5 to "N", and read pairs for which either end had less than 30 unambiguous base calls were removed. The latter step indirectly removes pairs containing mostly adaptor sequences. Read pairs were then aligned to the genome using STAR version 2.3.1 and gene counts were computed using HTSeq version 0.6.0.

Differential expression analyses comparing preimmunization sample counts from challenge-protected trial subjects to non-protected subjects were performed using DESeq2 version 1.28.0.

Gene Set Enrichment Analysis (GSEA) was performed on gene lists ranked by the DESeq2 Wald test statistic using the fgsea package version 1.14.0 in R. Gene sets used for GSEA were obtained through the tmod R package version 0.46.2 and the MSigDB Hallmark gene set download site (http://www.gsea-msigdb.org/gsea/msigdb/genesets.jsp?collection=H). Ingenuity Pathway Analysis (IPA) content version 62089861 was used to generate all IPA-based results. Receiver operating characteristic analyses were performed using the pROC package version 1.17.0.1 in R.

No custom algorithms or software packages were central to the research.

For manuscripts utilizing custom algorithms or software that are central to the research but not yet described in published literature, software must be made available to editors and reviewers. We strongly encourage code deposition in a community repository (e.g. GitHub). See the Nature Portfolio guidelines for submitting code & software for further information.

Data

Policy information about availability of data

All manuscripts must include a data availability statement. This statement should provide the following information, where applicable:

- Accession codes, unique identifiers, or web links for publicly available datasets
- A description of any restrictions on data availability
- For clinical datasets or third party data, please ensure that the statement adheres to our policy

Raw sequencing data for the MAL68 trial is publicly available through Sequence Read Archive BioProject PRJNA401870. Raw sequencing data for the CPS trial is publicly available at Sequence Read Archive BioProject PRJNA381264. Data for the IMRAS and BSPZV1 trials will be available through the ImmPort portal (immport.org).

Field-specific reporting

Please select the one below that is the best fit for your research. If you are not sure, read the appropriate sections before making your selection.

☒ Life sciences ☐ Behavioural & social sciences ☐ Ecological, evolutionary & environmental sciences

For a reference copy of the document with all sections, see nature.com/documents/nr-reporting-summary-flat.pdf

Life sciences study design

All studies must disclose on these points even when the disclosure is negative.

Sample size	No sample-size calculation was performed. We used the full complement of samples we could find from publicly-accessible trial data that fit our inclusion criteria as well as data obtained directly from collaborators (n=85). Previously published transcriptomic analyses using subsets of these samples were able to discern significant differences between trial groups; therefore, we anticipated that the total number of samples would be sufficient for identifying correlates of protection in our study.
Data exclusions	Principal Component Analysis (PCA) was used to identify outliers among transcriptomic samples: If a sample's first or second principal component value was more than three standard deviations from the corresponding principal component's mean, they were excluded from downstream analyses. One sample was identified as an outlier based on PCA.
Replication	The goal of our study was to identify transcriptomic correlates of protection that were consistent across clinical trials. We included all transcriptomic data that met our inclusion criteria in our effort to assess the reproducibility of correlates of protection, therefore our study design was based around discovering correlates with a high degree of reproducibility in independent cohorts.
Randomization	We analyzed transcriptomic data from four previously-published malaria vaccine trials that we did not conduct. For all four trials analyzed, allocation of participants into experimental group was randomized. However, randomization of the transcriptomic data we used for our analyses was not applicable: our analyses were based on comparisons between trial participants who were protected against malaria challenge following vaccination and those who were not.
Blinding	Blinding was not applicable. We aimed to make comparisons between data from protected and non-protected individuals and identify biological features associated with protection. This required knowledge of the protection outcome associated with each transcriptomic sample included in our study.

Reporting for specific materials, systems and methods

We require information from authors about some types of materials, experimental systems and methods used in many studies. Here, indicate whether each material, system or method listed is relevant to your study. If you are not sure if a list item applies to your research, read the appropriate section before selecting a response.

Materials & experimental systems

n/a	Involved in the study	
☒	☐	Antibodies
☒	☐	Eukaryotic cell lines
☒	☐	Palaeontology and archaeology
☒	☐	Animals and other organisms
☒	☐	Human research participants
☒	☐	Clinical data
☒	☐	Dual use research of concern

Methods

n/a	Involved in the study	
☒	☐	ChIP-seq
☒	☐	Flow cytometry
☒	☐	MRI-based neuroimaging
